# Age‐Associated Inflammatory Monocytes Are Increased in Menopausal Females and Reversed by Hormone Replacement Therapy

**DOI:** 10.1111/acel.70249

**Published:** 2025-10-09

**Authors:** R. P. H. De Maeyer, J. Sikora, O. V. Bracken, B. Shih, A. F. Lloyd, H. Peckham, K. Hollett, K. Abdelhamid, W. Cai, M. James, P. E. Pfeffer, M. Vukmanovic‐Stejic, A. N. Akbar, E. S. Chambers

**Affiliations:** ^1^ The Nuffield Department of Orthopaedics, Rheumatology and Musculoskeletal Science University of Oxford Oxford UK; ^2^ Centre for Immunobiology, Blizard Institute Queen Mary University of London London UK; ^3^ Department of Ageing, Rheumatology and Regenerative Medicine, Division of Medicine University College London London UK; ^4^ Department of Biomedical and Life Sciences Lancaster University Lancaster UK; ^5^ Cell Signalling and Immunology University of Dundee Dundee UK; ^6^ William Harvey Research Institute John Vane Science Centre, Charterhouse Square London UK; ^7^ Department of Infection and Immunity, Pears Institute University College London London UK

## Abstract

Biological sex is a crucial, but poorly understood variable in age‐related susceptibility to infection. Monocytes are important immune cells responsible for initiating and resolving inflammatory responses to infection. While changes in monocyte populations result in increased susceptibility to infection, there is limited research on the impact of age and sex on human monocyte phenotype and function. The aim of this work was to dissect the impact of increasing age and biological sex on human monocyte phenotype and function. Here, we show that older females have increased inflammatory intermediate and non‐classical monocytes compared to young. These monocyte subsets were the most inflammatory ex vivo, and their frequency correlated with markers of inflammageing. Proteomic analysis of sorted monocyte populations demonstrated that the three human monocyte subsets have largely distinct phenotypes. Key age‐associated protein pathways were identified, including complement cascade and phagocytosis. We confirmed the proteomics findings, showing that circulating C3 concentrations were reduced with age in females but not males. This decrease in complement in older females resulted in reduced monocyte phagocytosis. Crucially, we demonstrate that in peri/menopausal females, hormone replacement therapy (HRT) reversed this expansion in intermediate monocytes and decreased circulating CRP as compared to age‐matched controls. Importantly, peri/menopausal females on HRT had increased C3 serum concentrations and significant improvement in monocyte phagocytosis. The data presented here indicate the importance of menopause in aging monocyte phenotype and function. These data highlight the potential use of HRT in restoring monocyte function in females during aging and potentially improving anti‐pathogen immunity.

## Graphical abstract

Post‐menopausal females (> 64 years) have an increase in frequency of inflammatory monocyte populations with defective phagocytosis as compared to pre‐menopausal (< 40 years) females. Hormone Replacement Therapy (HRT) use in menopausal females (44–60 years) decreases the frequency of inflammatory monocytes and improves phagocytosis as compared to age‐matched controls.

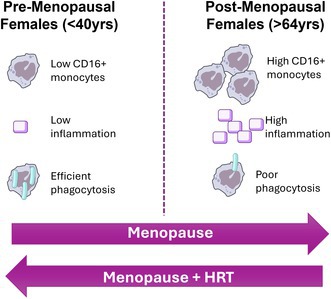

## 1 Introduction

Ageing is a global burden. Older people are living longer with increased morbidity and mortality from infections such as SARS‐CoV‐2 and influenza (Williamson et al. 2020; Fleming and Elliot 2005), malignancy (DePinho 2000), and reduced vaccine efficacy (Chambers et al. 2024) impacting upon health‐span. In addition to age, the biological sex of the individual greatly influences which infectious disease poses a higher risk of morbidity and mortality. Older males are known to be at increased risk of morbidity and mortality from SARS‐CoV‐2 (Williamson et al. 2020; Peckham et al. 2020) and community‐acquired pneumonia (CAP) (Almirall et al. 2017; Corica et al. 2022), whilst older females have increased morbidity and mortality from influenza (Klein et al. 2012) and non‐tuberculous mycobacterial pulmonary disease (NTM‐PD) (Mirsaeidi et al. 2014; Verma and Arora 2022). Menopause is a key life‐course event in females that leads to an increased incidence of inflammatory diseases such as asthma (Chowdhury et al. 2021; Triebner et al. 2016) and an increased risk of bacterial urinary tract infections (Flores‐Mireles et al. 2015).

Inflammageing is a phenomenon whereby circulating inflammatory mediators such as C Reactive protein (CRP), IL‐6, and IL‐8 (Ferrucci and Fabbri 2018) increase during ageing. This process underlies age‐associated pathologies including frailty (Samson et al. 2019), neurodegeneration (Weaver et al. 2002) and mortality (Bruunsgaard et al. 2003; Giovannini et al. 2011; Furman et al. 2017), and is linked to poor vaccine efficacy (Parmigiani et al. 2013; Fourati et al. 2016). Furthermore, monocyte‐derived inflammation negatively impacts antigen‐specific immunity in human skin (Chambers et al. 2021; Vukmanovic‐Stejic et al. 2018). Multiple factors contribute to the inflammageing phenomena; however, mononuclear phagocytes, and in particular monocytes, can contribute to the inflammatory milieu through Toll‐like receptor (TLR) binding to lipopolysaccharide (LPS) leaked into the circulation through increased gut permeability (Thevaranjan et al. 2018) and failure to resolve inflammation due to defects in efferocytosis (De Maeyer et al. 2020).

One of the key immune cells involved in coordinating immune responses to pathogens is the monocyte (Thevaranjan et al. 2018; Goritzka et al. 2015; Molony et al. 2017). Monocytes are circulating mononuclear phagocytes historically presumed to be solely precursor cells of tissue macrophages and dendritic cells. However, increasingly it has become apparent that monocytes are important innate immune cells in their own right and have important effector functions such as early pathogen recognition and initiation of inflammatory responses and phagocytosis (Mildner et al. 2024). Human monocytes can be separated into three populations based on their cell‐surface expression of CD14 and CD16, with CD14 + CD16‐ classical monocytes, CD14 + CD16+ intermediate monocytes and CD14‐CD16+ non‐classical monocytes (Patel et al. 2017). CD16+ monocytes have been shown to be expanded in the peripheral blood of older adults (Hearps et al. 2012; Metcalf et al. 2017). Highlighting their role in disease, it has previously been shown that older adults are at increased risk of Influenza infection due to an age‐related defect in RIG‐I signalling in monocytes (Molony et al. 2017). Furthermore, defective TNF production from monocytes of older people has also been associated with impaired pneumococcal immunity (Thevaranjan et al. 2018). Earlier studies using mixed cell populations have yielded conflicting data as to the effect of age on inflammatory cytokine production after stimulation in vitro (van Duin et al. 2007; Metcalf et al. 2015; Bruunsgaard et al. 1999). In addition, there is limited data as to the impact of the combination of age and sex on human monocyte function.

Indeed, the effect of biological sex in combination with age on immunosenescence is less well understood. Previous studies have shown that monocytes from males were more inflammatory in vitro as compared to females when stimulated with LPS (Beenakker et al. 2020), these data were not extrapolated by age. To date, there has been no study that assesses the impact of increasing age and biological sex on monocyte phenotype and function. Therefore, the aim of this paper is to dissect the impact of increasing age and biological sex on human monocyte phenotype and function.

## 2 Materials and Methods

### 2.1 Study Design

Ethics were approved by the Queen Mary Ethics of Research Committee reference number QMERC23.059 and by the University of Oxford Medical Sciences Interdivisional Research Ethics Committee (ref: R80191/RE001). Individuals 18 years or older were recruited into the study, and full written informed consent was obtained from each donor. 80 ml of peripheral blood was collected into sodium heparin tubes and processed as described below. Individuals were excluded from the study if they had a recent history (≤ 5 years) of neoplasia, inflammatory disorders which required immunosuppressive medication, were anaemic, or had a current pregnancy.

### 2.2 Whole Blood Flow Cytometry

Whole blood was labelled with the following cell surface antibodies: CD14 (HCD14), CD16 (3G8), CD19 (HIB19), CD20 (2H7), CD56 (HCD56), CCR2, CX3CR1, HLA‐DR (L243) and Zombie Green Fixable Viability Kit (Biolegend). Cells were incubated with antibodies for 45 min at 4°C. After incubation, red blood cells were removed using FACS Lysis buffer (BD Biosciences). Samples were subsequently washed twice in PBS and then assessed by flow cytometric analysis on a BD Fortessa (BD Biosciences) or on a Cytek Aurora (Cytek Biosciences).

FCS files of monocyte populations were exported using FlowJo Version X (BD Biosciences). Clustering and UMAP analysis of monocytes was performed using the R package ‘CATALYST’ (Nowicka et al. 2017). Briefly, fcs files are loaded into R studio, combined with the metadata, and prepared as a single cell experiment, which is then subject to clustering based on the expression of CD14, CD16, CCR2, HLA‐DR, CD86, SLAN, CLA, and CX3CR1. UMAPs were generated based on 4000 events from each sample.

### 2.3 Monocyte Isolation

Peripheral blood mononuclear cells were isolated by density centrifugation using Ficoll‐Paque (Amersham Biosciences). Monocytes were isolated by negative selection according to the manufacturer's instructions (Miltenyi Biotec).

### 2.4 Monocyte Cell Sorting

Negatively isolated monocytes were labelled with CD14 A700 (HCD14), CD16 BV786 (3G8), CD19 FITC (HIB19), CD20 FITC (2H7), CD56 FITC (HCD56) and HLA‐DR BV510 (L243) (Biolegend) for 45 min at 4°C. Cells were washed twice with PBS. Cells were sorted using the BD FACSAria III Cell Sorter (BD Biosciences). Monocytes were identified as being FITC lineage channel negative (CD3/CD19/CD20 and CD56) and HLA‐DR positive; within this population, three monocyte populations were sorted: classical (CD14^+^CD16^−^), intermediate (CD14^+^CD16^+^), and non‐classical (CD14^−^CD16^+^) (Figure S1). Sorted monocytes were either cultured in vitro or collected for proteomics analysis as described below.

### 2.5 Monocyte Culture

Negatively isolated or sorted monocytes were cultured in RPMI (Merck) supplemented with 10% FCS (Sigma‐Aldrich), 2 mM glutamine, and 100 U/mL penicillin/streptomycin (Thermo Fisher Scientific). Cells were cultured at 5% CO_2_ for 24 h. Supernatants were collected for cytokine analysis.

### 2.6 Phagocytosis Assay

100 μL whole blood obtained from K3EDTA vacutainers was added to a pre‐plated antibody cocktail containing CD14 APC‐Fire810 (63D3; Biolegend), CD16 eFluor450 (CB16; eBioscience), HLA‐DR BUV563 (L243; BD OptiBuild), CD115 BV711 (9‐4D2‐1E4; Biolegend), CD10 BV750 (HI10a; Biolegend), and Zombie‐NIR live/dead dye (Biolegend). Samples were challenged with 12.5 μL pHrodo red‐labelled *Escherichia coli* (E. coli) BioParticles (ThermoFisher, P35361) or PBS control and left for 30 min at 37°C. 500 μL Fix/Lyse (eBioscience) solution was added to stop phagocytosis and lyse red blood cells. Samples were washed and acquired on a Sony ID7000 Spectral Analyser (Sony Biotechnology).

### 2.7 Supernatant Cytokine Assessment

Cytokine concentrations in culture supernatants were assessed by cytometric bead array (BD Biosciences) according to the manufacturer's protocol. Samples were analyzed using a Novocyte flow cytometer (Agilent).

### 2.8 Proteomics

Sorted monocytes were collected into eppendorfs containing RPMI with 1% FCS. Subsequently, the pellets were washed in cold PBS, and then pellets were snap frozen on dry and ice and stored at −80°C. Samples were lysed in 400 μL lysis buffer (5% SDS, 10 mM TCEP, 50 mM TEAB in highly pure H_2_O) and shaken at RT for 5 min at 1000 rpm, followed by boiling at 95°C for 5 min at 500 rpm. Samples were then shaken again at RT for 5 min at 1000 rpm and sonicated for 15 cycles of 30s on/30s off with a BioRuptor (Diagenode) before Benzonase was added to each sample and incubated at 37°C for 15 min to digest DNA. Next, samples were alkylated with 20 mM iodoacetamide for 1 h at 22°C. Protein concentration was determined using an EZQ protein quantification kit (Invitrogen) following the manufacturer's instructions. Protein isolation and cleanup were performed using S‐TRAP (Protifi) columns before digestion with trypsin at a 1:20 ratio (enzyme:protein) for 2 h at 47°C, and digested peptides were eluted from S‐TRAP columns using 50 mM ammonium bicarbonate, followed by 0.2% aqueous formic acid and 50% aqueous acetonitrile containing 0.2% formic acid and dried overnight. Peptides were resuspended in 1% formic acid and injected onto a nanoscale C18 reverse‐phase chromatography system (UltiMate 3000 RSLC nano, Thermo Scientific) and electrosprayed into an Orbitrap Exploris 480 Mass Spectrometer (Thermo Fisher).

Raw files are processed through Spectronaut 18 to identify and quantify proteins, which are then run through Perseus (v2.0.11), which converts the intensity values of each protein into copy numbers. Additionally, Spectronaut files were analyzed using mass spectrometry downstream analysis pipeline (MS‐DAP v 1.2.1).

### 2.9 Serum Protein Analysis

The serum levels of CRP, C3, and C4 were measured using sandwich ELISA kits following the manufacturer's instructions. Dilutions were made as needed to ensure that the readings fell within the ranges of the assays. The concentration of CRP was measured using the DuoSet Human C‐Reactive Protein/CRP DuoSet ELISA kit (Cat. No. DY1707) from R&D Systems (Minneapolis, MN, USA). The concentrations of C3 (Cat. No. ab108822) and C4 (Cat. No. ab108824) were determined using Abcam kits (Abcam, Cambridge, UK). The sensitivities of these kits were approximately 994 μg/mL for C3 and 0.53 μg/mL for C4. The precision (intra‐assay and inter‐assay) for all the assays was approximately 10%.

### 2.10 Statistical Analysis

Statistical analysis was performed using Prism version 10.0.0 (GraphPad). Data were assessed for normality, and the subsequent appropriate two‐sided statistical test was performed as indicated in the figure legend. For the correlations, Pearson's correlation was performed in Prism.

## 3 Results

### 3.1 Increased Frequency of CD16 Expressing Monocytes in Older Adults

**FIGURE 1:**
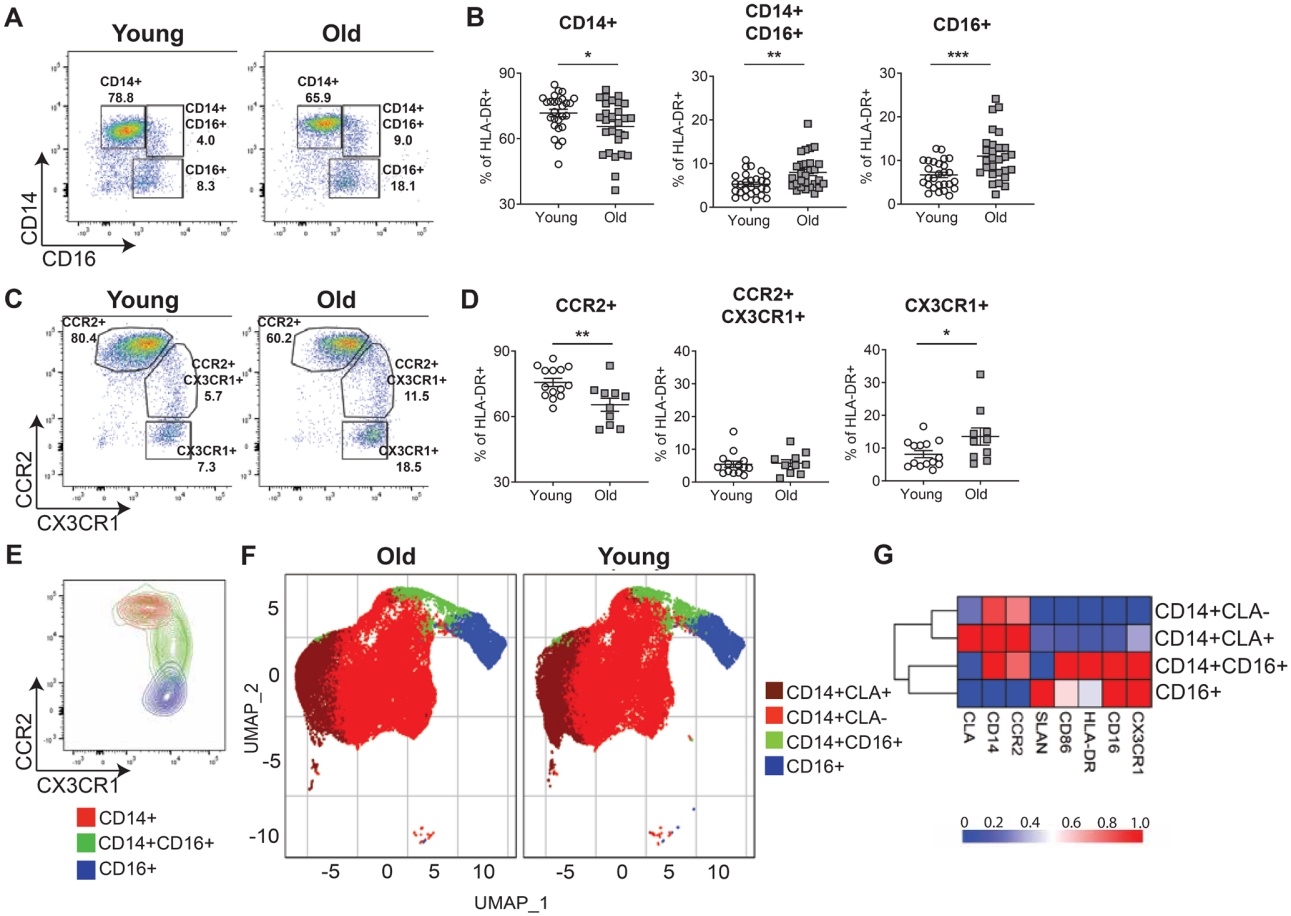
Increased frequency of non‐classical and intermediate monocytes in older adults. Whole blood was assessed by flow cytometry, and monocytes were identified as being Lineage negative HLA‐DR+. (A) representative flow plots, and (B) cumulative data showing the frequency of CD14+ (classical), CD14 + CD16+ (DP; intermediate), and CD16+ (non‐classical) monocytes; and (C) representative flow plots, and (D) cumulative data showing the frequency of CCR2+ (classical), CX3CR1 + CCR2+ (DP; intermediate), and CX3CR1+ (non‐classical) monocytes in the peripheral blood of young (white; < 40 years) and old (grey; ≥ 65 years) donors. (E) a representative image showing an overlay plot of CD14+ (red), CD14 + CD16+ (green), and CD16+ (blue) monocytes according to the expression of CCR2 and CX3CR1. Bioinformatic analysis was performed on monocytes and analyzed based upon the monocyte markers SLAN, CLA, CCR2, CD14, CD16, CD86, HLA‐DR, and CX3CR1. (F) UMAP analysis of monocytes from young and old monocytes; and (G) a heatmap of the marker expression in the four clusters. (B and D) assessed by *t*‐test. *p < 0.05; **p < 0.01; ***p < 0.001.

To assess the phenotype of monocytes in young (< 40 years) and older adults (≥ 65 years), whole peripheral blood was labelled for flow cytometric analysis, and monocytes were identified from live single cells as being lineage negative (CD3, CD19, CD20 and CD56) and HLA‐DR+ (Figure S1). Monocyte subsets were identified based on the expression of CD14 and CD16; classical monocytes as CD14 + CD16‐ (CD14+), intermediates as CD14 + CD16+ and non‐classicals as CD14‐CD16+ (CD16+). Older adults (mean age 73.1; 17 females and 14 males) had a significant increase in intermediate and non‐classical monocytes compared to younger adults (mean age 29.2; 26 females and 17 males) (Figure 1A,B). This was further examined using alternative markers for monocytes CCR2 and CX3CR1, and in line with earlier work from Patel et al. (2017), it was confirmed that there was a significant increase in the frequency of non‐classical monocytes (CCR2‐CX3CR1+) in older adults as compared to young (Figure 1C,D). As expected, the expression of CCR2 and CX3CR1 correlated with CD14 and CD16 (Figure 1E), demonstrating that non‐classical and intermediate monocytes were increased in older adults as compared to young. Unsupervised analysis of the data was performed, where cells were clustered using the FlowSOM algorithm, which identified four unique clusters: CLA + CD14+, CLA‐CD14+, CD14 + CD16+, and CD16+. These clusters were visualised via a UMAP (Figure 1F,G). There was no difference in the surface expression of proteins studied (CLA, CD14, CCR2, CD86, CD16, SLAN, CX3CR1, and HLA‐DR) according to age (Figure S2), suggesting the age‐related change in monocyte subset quantification is likely due to increased monocyte differentiation. Collectively, this data shows that the proportion of monocyte subsets significantly changes in aging.

### 3.2 CD16 Expressing Monocytes Are More Inflammatory and Correlate With Inflammageing

**FIGURE 2:**
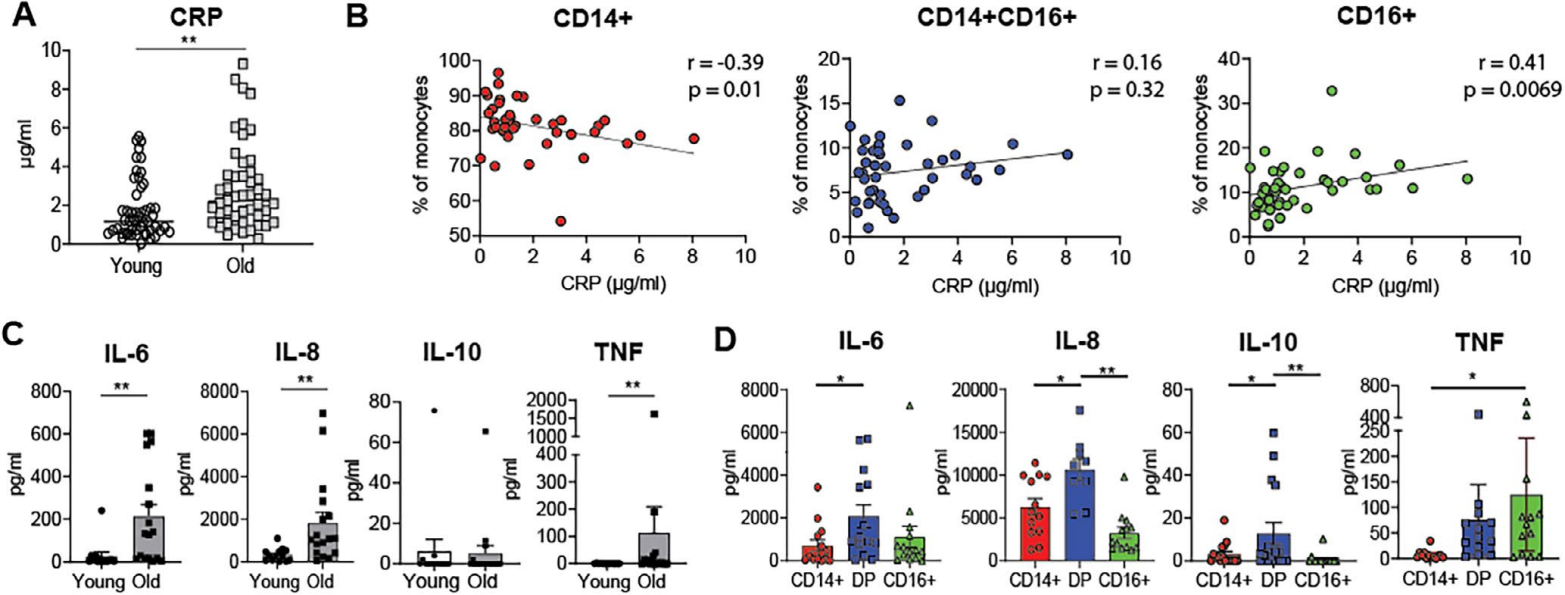
CD16+ monocytes are more inflammatory and correlate with C reactive protein, a marker of inflammageing. Serum C Reactive Protein (CRP) concentrations were assessed in biobanked serum samples by ELISA (A) Serum CRP concentrations in young (white; *n* = 50) and old (grey; *n* = 48) donors and (B) Serum CRP correlated with the frequency of monocyte populations in the peripheral blood of the same individual. (C) Monocytes were isolated from the peripheral blood and cultured (unstimulated) for 24 h; supernatants were collected and assessed by cytometric bead array cumulative cytokine production from young (white; *n* = 13) and old (grey; *n* = 17) donors. (D) Cumulative data of monocytes sorted into three populations: classical (CD14+), intermediate (CD14 + CD16+), and non‐classical (CD16+) and cultured (unstimulated) for 24 h. Cytokine production after 24 h from CD14+ (red; *n* = 13), CD14 + CD16+ (green; *n* = 13), and CD16+ (blue; *n* = 13). (A) assessed by Kruskal‐Wallis test with Dunn's multiple comparisons test and (B) assessed by Pearson's correlation test. (C and D) assessed by Mann Whitney. (E) assessed by One‐way ANOVA with Tukey's multiple comparison test. *p < 0.05; **p < 0.01.

Inflammageing is defined as low‐grade chronic inflammation characterised by elevated circulating inflammatory proteins such as C Reactive Protein (CRP) (Ferrucci and Fabbri 2018; Franceschi et al. 2017), which is observed during ageing. As expected, we observed a significant increase in serum CRP in older adults as compared to young (Figure 2A). To determine if the increased proportion of CD16‐expressing monocytes in older adults corresponds to increased markers of inflammageing, CRP was correlated with the frequency of monocyte populations. It was observed that there was a significant positive correlation between serum CRP concentrations and the frequency of CD16+ non‐classical monocytes (Figure 2B), with the individuals who had the most CD16+ non‐classical monocytes having the highest serum CRP concentration. Furthermore, a significant negative correlation was observed between CRP serum concentrations and the frequency of CD14+ monocytes.

Alongside higher levels of CRP, inflammageing is also associated with increased levels of IL‐6, IL‐1β, and TNF (Ferrucci and Fabbri 2018; Franceschi et al. 2017). To investigate the effect of inflammatory cytokine expression, monocytes were isolated from young and old donors and cultured for 24 h without additional stimulation (unstimulated). Cytokine expression in the supernatant was assessed by cytometric bead array, and it was observed that monocytes from older individuals produced significantly more IL‐6, IL‐8, and TNF, but not IL‐10, than monocytes from younger individuals (Figure 2C). To determine which monocyte subsets from older adults make more inflammatory cytokines, monocyte subsets were sorted by FACs and cultured at the same cell concentration, separately, for 24 h unstimulated. CD16 + CD14+ monocytes expressed significantly more IL‐6, IL‐8, and IL‐10 as compared to the other monocyte subsets (Figure 2D). However, CD16+ non‐classical monocytes secreted significantly more TNF as compared to CD14+ monocytes (Figure 2D). No significant difference in cytokine production by the individual monocyte populations was observed with age (Figure S3). This indicates that the inflammageing process is linked to an increased frequency of intermediate and non‐classical monocytes, which are increased in frequency in older individuals. This increased frequency of intermediate and non‐classical monocytes is associated with higher production of inflammatory cytokines, as opposed to an increased capacity for cytokine production by the individual monocyte cells with age.

### 3.3 The Proteome of the Three Different Monocyte Populations Is Significantly Different, Indicating Distinct Functionality

Due to the effects of age on monocyte phenotype and function and the significant differences observed, proteomic analysis was performed on monocytes from young and old donors, and to enable deeper phenotyping of the effect of age on monocyte subsets, we performed proteomic analysis of the individual monocyte populations (CD14+ classical; CD14 + CD16+ intermediate; CD16+ non‐classical) from 8 young and 8 old donors. Protein expression is the main mechanistic indicator of function and can therefore provide detailed insight into functional differences between monocyte populations. The CD14 and CD16 expression levels from the proteomic data were utilized to confirm that their levels correspond to the CD14 and CD16 labeling from flow cytometry. PCA analysis showed distinct proteomes of the three monocyte populations (Figure S4A), and protein expression of CD14 and CD16 was observed in the expected populations (Figure S4B). The number of proteins quantified between the three different monocyte populations was also not statistically different (Figure S4C).

**FIGURE 3:**
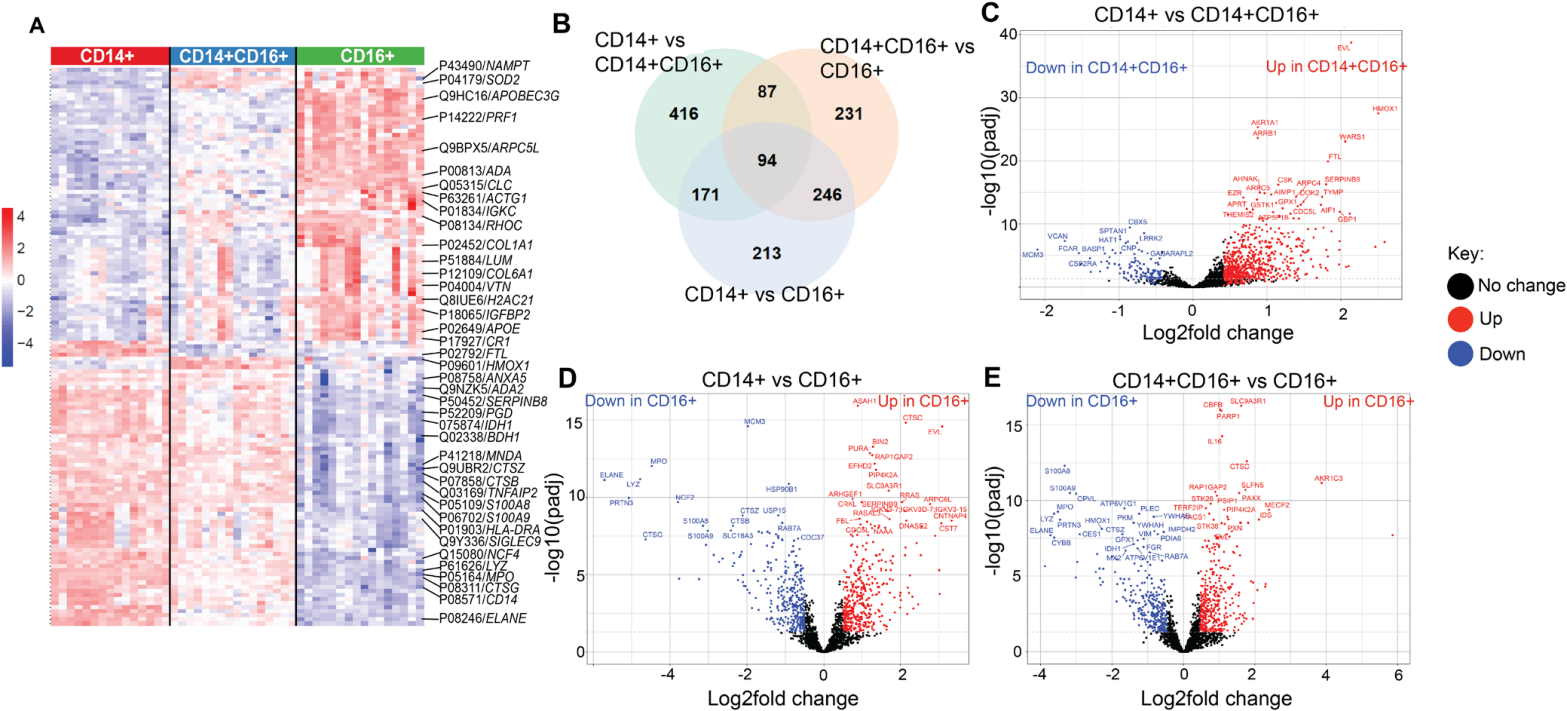
Proteomic composition of the three different monocyte populations is significantly different. Monocytes were isolated from the peripheral blood and sorted ex vivo into three populations: classical (CD14+; red, *n* = 16), intermediate (CD14 + CD16+; blue, *n* = 16), and non‐classical (CD16+; green, *n* = 16). Proteomics analysis was performed on the ex vivo cell pellets. (A) heatmap of significantly differentiated protein copy numbers, including highlighted proteins (with gene names), is shown. Proteomics was analyzed by the package MSDAP to assess over protein expression between the three different monocyte subsets. (B) Venn diagram showing differentially expressed proteins in the three monocyte subsets and significantly differentially expressed proteins in (C) CD14+ vs. CD14 + CD16+; (D) CD14+ vs. CD16+; and (E) CD14 + CD16+ vs. CD16 + .

The top significantly differentially expressed proteins according to monocyte population (transitioning from CD14+, CD14 + CD16+, through to CD16+) were assessed, and it was observed that there were significant proteomic differences between the three different monocyte populations (Figure 3A; Table S1). The package MS‐DAP was used to quantify protein intensity in monocyte populations. Differential expression analysis using the algorithm MSqRob was employed to quantify proteomic differences between the subsets. The differentially expressed proteins in each comparison were visualised using a Venn diagram (Figure 3B), revealing 416 uniquely expressed proteins in CD14+, as compared to CD14 + CD16+, and 231 uniquely expressed proteins in CD14 + CD16+ as compared to CD16+, and 213 uniquely expressed proteins in CD16+ as compared to CD14+ (Figure 3B). A full list of differentially regulated proteins identified in the heatmap can be found in Table S2. To visualise the differences between these contrasts, we used volcano plots to view the top 25 upregulated and downregulated proteins, further showing the unique signatures between the different subsets (Figure 3C–E). There was significantly higher expression of HMOX1, EVL, FTL, whilst there was significantly less MCM3, VCAN, CSF2RA in CD14 + CD16+ as compared to CD14+ monocytes (Figure 3C). Expression of CTSC, EVL, ASAH1, BIN2 was increased, whilst there was significantly less MPO, LYZ, ELANE, S100A8, and S100A9 in CD16+ as compared to CD14+ monocytes (Figure 3D). Similar downregulated proteins were observed in CD16+ monocytes as compared to CD14 + CD16+ (Figure 3E). However, there were significant differences in upregulated proteins in CD16+ as compared to CD14 + CD16+ with a significant increase in IL‐16, CBFB, SLC9A3R1, and PARP1 (Figure 3E). Given the differences in monocyte subset proportions with age and the distinct proteomic differences of the three different monocyte populations, this data support the hypothesis that there will be an overall alteration in monocyte functionality with increasing age.

### 3.4 The Proteome of Monocytes Is Significantly Altered With Age

**FIGURE 4:**
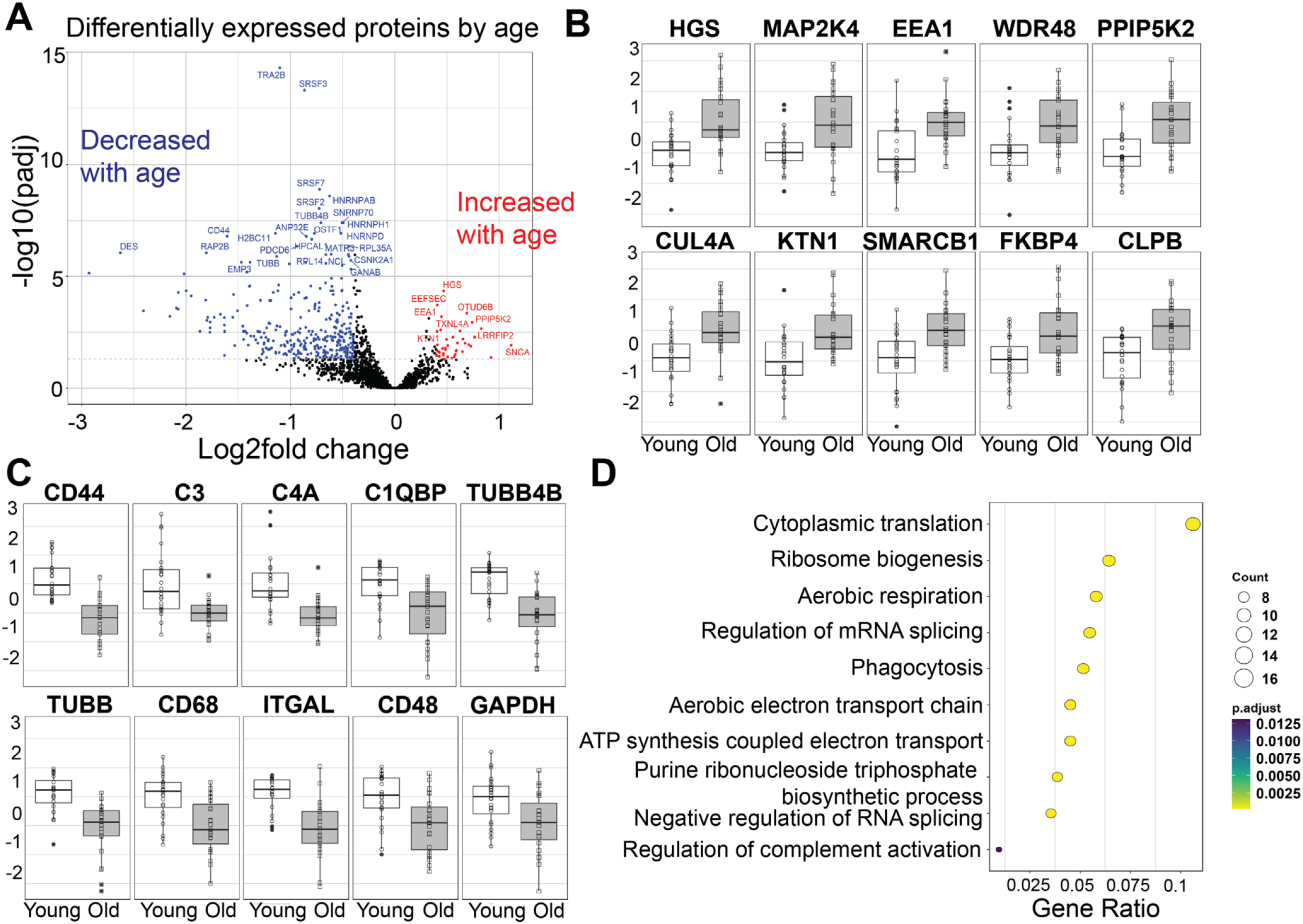
Older monocytes have significantly altered proteomes, including reduced phagocytic proteins. Monocytes were isolated from the peripheral blood and sorted into three populations: classical (CD14+), intermediate (CD14 + CD16+), and non‐classical (CD16+). Proteomics analysis was performed on the ex vivo cell pellets from young (white; *n* = 8) and old (grey; *n* = 8) donors. (A) Volcano plot showing the differentially regulated proteins. (B) Significantly increased proteins in old monocytes as compared to young. (C) Significantly decreased proteins in old monocytes as compared to young. (D) Pathway analysis of pathways significantly decreased in old monocytes as compared to young.

There was a subtle but striking separation of the monocyte subsets on PCA analysis by age (Figure S4D). Subsequently, the proteomic dataset was analyzed to determine which proteins were significantly altered with age in all monocyte populations. It was determined that there were 369 proteins whose expression was significantly altered with age; the vast majority of these were downregulated in old as compared to young (Figure 4A; Table S2). Proteins significantly increased in older monocytes included those involved in endocytosis (HGS, EEA1), intracellular signaling (MAP2K4, PPIP5K2), ubiquitination (WDR48, CUL4A), intracellular organelle motility (KTN1), chromatin remodeling (SMARCB1), and metabolism (CLPB) (Figure 4B; the full list of significantly changed proteins can be seen in Table S3). Proteins that were significantly decreased in older monocytes as compared to young included complement proteins (C3, C4 and C1QBP), proteins involved in migration (CD44, TUBB4B, TUBB and ITGAL), metabolism (GAPDH) and scavenger receptors (CD68 and CD48) (Figure 4C).

To dissect which pathways were specifically decreased with age, pathway analysis using GO terms was performed. There was a significant downregulation in metabolic pathways such as aerobic respiration, purine ribonucleoside triphosphate biosynthetic process, and phagocytosis‐associated pathways such as phagocytosis and regulation of the complement cascade (Figure 4D). Indeed, when phagocytosis proteins were assessed individually, they were all significantly downregulated in older monocyte populations as compared to young (Figure S5). Collectively, this proteomic data proposes that older monocytes have a significantly altered proteome with reduced metabolic and phagocytic pathways.

### 3.5 Monocyte Phenotype and Phagocytic Function Is Significantly Altered in Older Females

**FIGURE 5:**
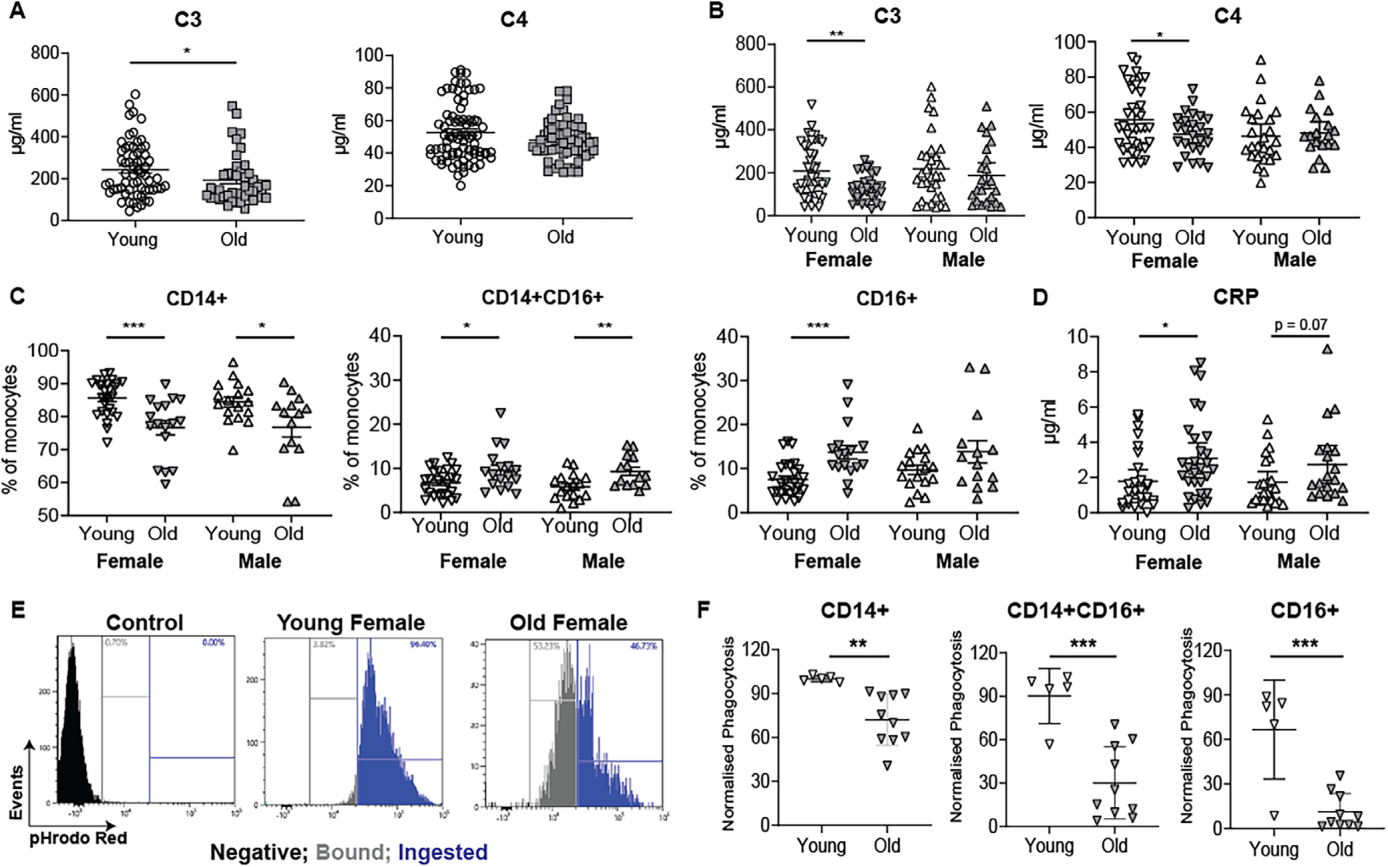
Concentration of complement proteins and the capacity for phagocytosis are reduced in older females. Serum samples were assessed for C3 and C4 concentrations by ELISA (A) serum C3 and C4 concentrations in young (white; *n* = 62) and old (grey; *n* = 43) donors. (B) Serum C3 and C4 (young female *n* = 43; old female *n* = 30; young male *n* = 31; old male *n* = 24) and (C) proportion of monocytes (young female *n* = 26; old female *n* = 17; young male *n* = 17; old male *n* = 14) and (D) serum CRP concentrations (young female *n* = 27; old female *n* = 29; young male *n* = 23; old male *n* = 19) split according to age (young, < 40 years., white; old, ≥ 65 years., grey) and sex (males, upwards triangles; females, downwards triangles). Whole blood phagocytosis assay was performed (E) representative dot plot with control gate (black), bound gate (grey), and ingested gate (blue) and cumulative data in CD14+, CD14 + CD16+, and CD16+ and % of cells ingested BioParticles as normalized to young CD14+ monocytes in (F) female individuals. (B–D and F) assessed by unpaired t test. *p < 0.05; **p < 0.01; ***p < 0.001.

As phagocytosis and complement pathways were significantly reduced in older monocytes (Figure 4D), the expression of complement proteins in serum was investigated to determine if this decrease was also seen systemically. Biobanked serum from young and old donors was assessed for C3 and C4 concentrations by ELISA. There was significantly less C3 but not C4 in older donors as compared to young (Figure 5A). Donors recruited in this study had a female bias and as previous data suggest there may be differences in the function of monocytes according to biological sex (Beenakker et al. 2020). Thus, we separated the complement data according to biological sex and observed that older females had a significant reduction in C3 and C4 as compared to young females (Figure 5B). This was not observed in males. In addition, there was a significant inverse correlation between serum C3 concentration and age in females but not in males (Figure S6A).

Given the striking difference in serum C3 concentrations in females with age, we analysed monocyte proportions according to age and sex to assess covariance. We observed a decrease in CD14 + monocytes in both males and females with age, but the difference was more significant in females (Figure 5C). There was a significant increase in intermediate monocytes with age in both sexes; however, an increase in CD16+ monocytes was only observed in older females (Figure 5C). Interestingly, when complement receptors (CD11b, CD11c, and CD18) were assessed in the proteomic dataset, it was observed that there was significantly higher expression on CD14+ monocytes as compared to CD14 + CD16+ and CD16+ monocytes (Figure S7). No significant differences in complement receptor expression were observed with age (data not shown). Serum CRP was found to be significantly increased in females with age, whilst a trend was observed in males (Figure 5D). When the correlation between CRP and monocyte proportions was assessed, CD16+ monocytes positively and CD14+ monocytes negatively correlated in females but not males (Figure S8), showing similar significance in females as observed in Figure 2B.

To determine if the change in monocyte proportions and circulating complement in older females corresponded to functional deficiency, a whole blood phagocytosis assay was performed. Whole blood was incubated with fluorescently labelled *E. coli* BioParticles and phagocytosis was assessed 30 min after the incubation. Samples displayed an overall shift in fluorescence intensity over unstimulated controls due to the BioParticles binding to cells but not being internalised to fully activate the pH‐reactive dye. As such, we have gated both bound and internalised events (Figure 5E). It was observed that there was significantly less internalisation of *E. coli* in older females as compared to young females (Figure 5E,F). There was no significant effect of age in male donors (Figure S6B), demonstrating that this was a female age‐associated effect.

Collectively, this data shows that there is an age‐associated defect in monocytes from females with age. This results in a significant reduction in monocyte phagocytosis in females, but not males, due to reduced circulating C3.

### 3.6 HRT Alters Monocyte Phenotype and Improves Function in Peri−/Menopausal Females

**FIGURE 6:**
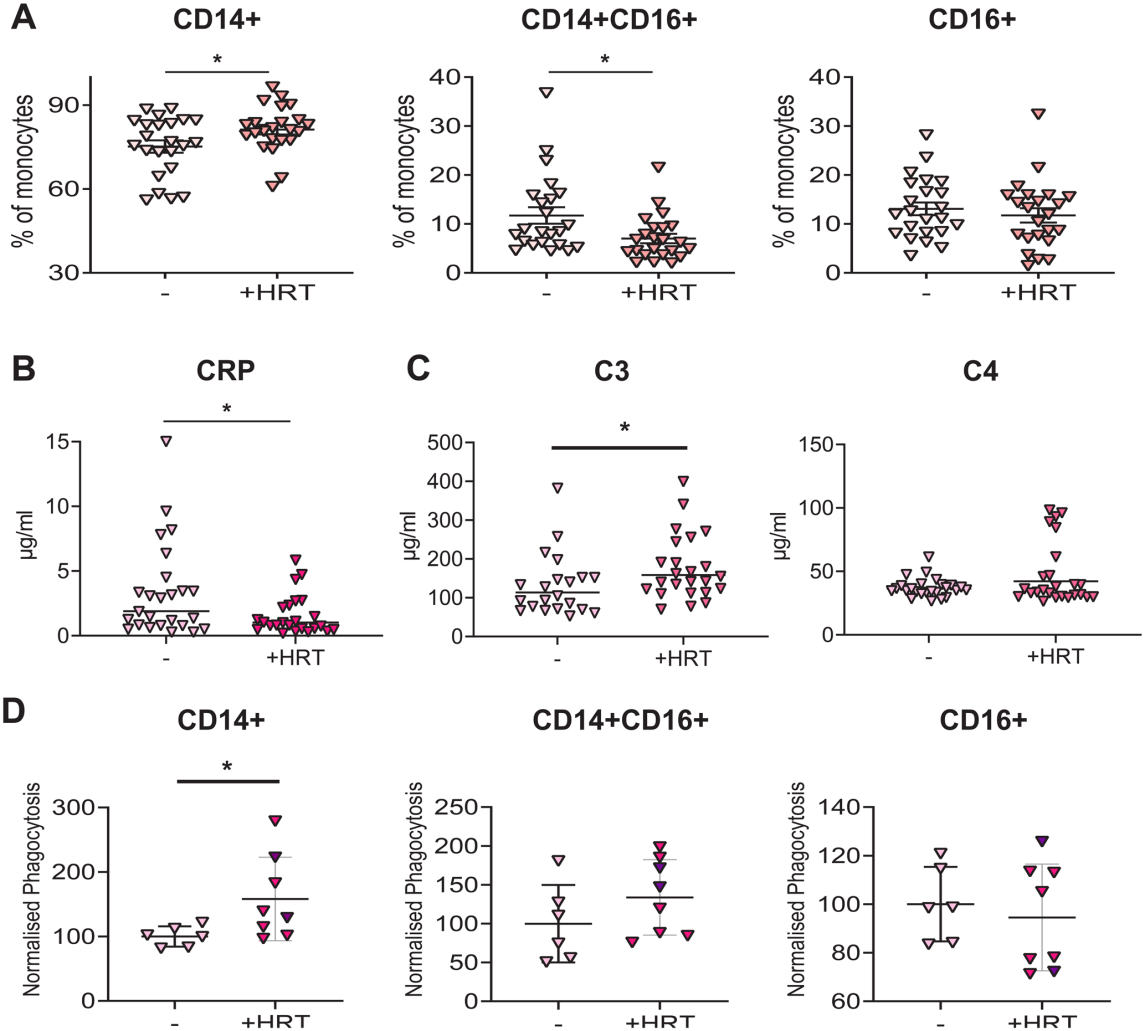
HRT rescues female age‐associated intermediate monocyte expansion and phagocytosis activity. Whole blood and serum were collected from peri‐/menopausal females who were either taking or not taking Hormone Replacement Therapy (+HRT). (A) Whole blood was assessed by flow cytometry, and monocytes were identified as being Lineage negative HLA‐DR+, frequency of CD14+ (classical), CD14 + CD16+ (DP; intermediate), and CD16+ (non‐classical) monocytes (*n* = 25 each group). (B) CRP serum concentrations measured by ELISA (*n* = 25 each group). (C) Serum C3 and C4 concentrations measured by ELISA (*n* = 25 each group). (D) cumulative whole blood phagocytosis data in CD14+, CD14 + CD16+, and CD16+ (no HRT *n* = 6; HRT *n* = 8). Donors who received progesterone via the coil are indicated in dark purple (*n* = 2). (A–D) assessed by *t*‐test. * = *p* < 0.05.

One key life‐course event which occurs in females but not males is the menopause. Menopause is associated with the reduction and eventual loss in female sex hormones from the ovaries. Many women counteract this loss of female sex hormones via the use of hormone replacement therapy (HRT) (Tang et al. 2025). To assess whether the loss of sex hormones as a result of menopause leads to the change in monocyte phenotype in older females, peri/menopausal women on HRT were recruited and assessed for monocyte phenotype by flow cytometry, table of donor characteristics found in Table 1. Females who were on HRT had significantly fewer CD14 + CD16+ intermediate monocytes and CD14+ classical monocytes as compared to age‐matched controls (Figure 6A), although no significant difference was observed in the CD16+ non‐classical monocyte population. Menopause and the loss of sex hormones seem to be a key drivers in the change in monocyte proportions, as the older women taking HRT have a similar frequency of CD14 + CD16+ monocytes as compared to young females. As we previously identified a correlation between inflammatory monocytes and serum CRP, serum CRP levels were assessed in the two female middle‐aged cohorts. A significant decrease in CRP concentrations was observed in those individuals taking HRT as compared to age‐matched controls (Figure 6B). HRT is often prescribed differently for each person; all females in this study received the combination of oestrogen and progesterone. Within our cohort, Oestrogen *(17‐β Oestradiol)* was either administered orally or cutaneously. Whilst progesterone was either administered orally (either as Utrogestan or norethisterone; cyclically) or locally in the uterus via the progesterone‐producing coil (continuous exposure). To investigate whether the route of administration impacted serum CRP or complement proteins or the frequency of monocytes, the data was separated by route of administration of the female sex hormone. Of the donors who declared the route of administration for oestrogen (2 donors did not provide this information), the majority received oestrogen via the skin route, and only three donors took oestrogen orally. We observed no significant difference in serum CRP, C3, or C4 or the frequency of monocyte populations when the data was separated by route of oestrogen administration (Figure S9A–C). For progesterone, whilst the majority received progesterone via the oral route, eight of the donors received progesterone locally in the uterus via the progesterone‐producing coil. It was observed that there was a trend towards reduced circulating CRP in those who received progesterone via the coil (Figure S9D; *p* = 0.06). There was also a trend towards an increase in circulating C3 in those who received progesterone via the coil (Figure S9E; *p* = 0.07). There was no significant difference in circulating C4 concentrations or the frequency of the monocyte populations between those who received progesterone orally or via the coil (Figure S9E,F).

**TABLE 1:** Middle‐aged female donor characteristics.

	Age‐matched controls	+ HRT
Sex	24 Female	24 Female
Age	50 (43–61)	51 (44–60)
Ethnicity	15 Caucasian (British)	14 Caucasian (British)
	4 Caucasian (Other)	7 Caucasian (Other)
	1 Mixed	0 Mixed
	1 South Asian	0 South Asian
	1 East Asian	0 East Asian
	2 Black (British)	1 Black (British)
	0 Black (Caribbean)	1 Black (Caribbean)
	0 did not declare	1 Did not declare
	*n* = 24	*n* = 24

*Note:* Age presented as median with range.

This raises the question of the functionality of monocytes during ageing and how HRT is HRT affecting these functions. The concentration of C3 and C4 was assessed in the serum of peri‐/menopausal females with and without HRT. It was observed that there was significantly more circulating C3 in the serum of females on HRT as compared to age‐matched controls (Figure 6C). Finally, we observed that peri‐/menopausal females on HRT had a significantly increased phagocytosis as compared to age‐matched controls (Figure 6D).

These experiments show that menopause is associated with a reduction in C3 and phagocytosis, with an associated increase in CD16+ monocytes, which may be reversed by HRT.

## 4 Discussion

This is the first proteomic and functional analysis to assess the impact of age and sex on the phenotype and function of monocytes in humans. We, like others, observed a relative expansion of intermediate and non‐classical monocyte subsets at the expense of classical monocytes with age (Hearps et al. 2012; Nyugen et al. 2010; Cao et al. 2022). We observed a particularly pronounced phenotype in older females compared to males. We also show that these expanded subsets are more inflammatory when left unchallenged ex vivo, and that a shift away from classical to non‐classical monocytes correlates with markers of inflammageing such as systemic CRP. Proteomic analysis of monocyte populations identified a significant downward shift in protein expression with age, especially in pathways involved in metabolism, complement activation, and phagocytosis. These findings were validated by a significant reduction of serum C3 concentration and the reduced phagocytic capacity of monocytes in older females. Finally, HRT use in peri–/menopausal women rescues the female age‐associated alterations in monocytes, increasing circulating C3 and enhancing monocyte phagocytosis. These data suggest that intermediate and non‐classical monocytes inherently underlie systemic inflammatory features. Thus, their expansion in older people, in particular older women, might be a driver of inflammageing, which can be targeted using HRT.

The change to monocyte subset proportions in age has been assessed previously, and our data replicates other datasets (Hearps et al. 2012; Metcalf et al. 2015) showing older adults have an increase in CD16+ monocytes. Although some studies have not observed these differences, we posit this could be a result of the effect of other variables like biological sex (Reitsema et al. 2024). To our knowledge, only one study assessing monocyte phenotype with age extrapolated the data based upon biological sex (Tampe et al. 2024). However, we observed that older females have a more significant increase in CD16+ monocytes, a population that has been linked to inflammation and inflammatory disease states (Mukherjee et al. 2015). Understanding the effect of biological sex and sex hormones on immunity has become an important and necessary research area. Recently, it has been observed that oestrogen can increase class‐switched B cells in females on an XX chromosome background (Peckham et al. 2025). Whilst testosterone, administered via gender‐reaffirming hormone therapy in transgender males, can make monocytes more inflammatory with increased cytokine production. This study is the first data set to assess the impact of age and biological sex on monocyte phenotype and function (Lakshmikanth et al. 2024). Although we did not observe any difference with sex regarding inflammatory cytokine production, further research may be needed to determine at what age testosterone and oestrogen have the biggest influence on inflammatory cytokine production from monocytes. It is clear from this data that the menopause has a dramatic impact upon immunity in females, and, as such, this is an important area of research to evaluate further.

We show that older monocytes make significantly more inflammatory cytokines compared to younger monocytes when cultured for 24 h in the absence of stimulation. This increase in inflammatory cytokine production is explained by the increase in the frequency of CD16+ monocytes in older people, rather than the per cell expression of pro‐inflammatory cytokines. This is in line with earlier studies which showed that there was no difference in sorted monocyte population inflammatory cytokine production with age and speaks to the importance of correcting imbalanced monocyte populations with age/sex or in disease contexts (Metcalf et al. 2017) In contrast, we did observe cellular dysfunction in terms of phagocytosis in older females. Previous studies into aged mouse models have observed that macrophages from older mice have reduced phagocytosis (Swift et al. 2001; Linehan et al. 2014). More recently, we observed that macrophages in a skin blister model have reduced efferocytosis in older adults (De Maeyer et al. 2020). Ours is the first study to look at the impact of age and sex on monocyte phagocytosis, in which we observed reduced phagocytosis in older compared to younger females, with males showing no age‐dependent change in phagocytic function. These data provide a hypothesis for why older females are more susceptible to bacterial infections such as NTM and bacterial UTIs.

Crucially, we have shown the potential importance of HRT in the restoration of monocyte subset proportion and function in older females. HRT has been transformative in improving the lives of menopausal and perimenopausal women by improving the clinical symptoms that some women suffer from during this time (Lobo 2017). Our study is the first to show the potential impact that this hormone replacement has on the function of the ageing female immune system. We show that HRT may reverse the age‐related increase in inflammatory monocyte subsets and restore monocyte phagocytic functionality. Although we age‐matched our middle‐aged donors, we were not able to determine that the individuals were at the same stage of ovarian ageing. Thus, to fully validate these effects of HRT, a study would need to be carried out in the same individuals pre‐ and post‐HRT. We also observed some trends in circulating CRP and C3 associated with the route of administration of HRT; however, due to the small sample size in some of the groups, this analysis would need to be repeated on a larger dataset to determine if the route of administration is important for immunophenotyping of the immune cells. Whilst there is much discussion on the benefits and risks of HRT (Almirall et al. 2017; Hodis and Mack 2022; Gu et al. 2024; Mehta et al. 2021), there is currently little evidence supporting how, at the molecular level at least, these drugs could be crucial in improving the immune health span of females during ageing. This study should serve as a call for more dedicated studies looking at HRT and lowered infection risk in older women.

To conclude, we have shown that being older and female dramatically impacts upon monocyte phenotype and function, with increased inflammatory CD16+ monocytes and reduced phagocytosis contributing to inflammaging and, as such, decreased health‐span. Collectively, these data highlight the importance of studying age and sex as biological variables within experiments and provide evidence that HRT could be instrumental in modulating age‐dependent inflammation and the complications thereof.

## Author Contributions

E.S.C. designed the study, performed experiments, analyzed data, and wrote the first draft. R.P.H.D.M. performed experiments, analyzed data, and wrote the first draft. J.S., K.A., K.H., and W.C. performed experiments. M.J., H.P., and P.E.P. were involved in sample recruitment and collection. M.V.‐S. and A.N.A. were involved with the early study design. O.V.B. and B.S. performed the proteomic bioinformatic analysis. A.F.L. performed the proteomic analysis. All co‐authors read the final version of the manuscript.

## Conflicts of Interest

The authors declare no conflicts of interest.

## Supporting information


**Figure S1:** Example gating strategy to identify monocytes in whole blood. Figure shows representative gating strategy to identify monocytes in whole blood that was assessed by flow cytometry. Leukocytes were identified as being CD45+; subsequently, single cells were identified using SSc‐A and SSc‐H. Next lineage negative (CD3, CD19, CD20, and CD56) cells were identified then HLA‐DR+ cells were selected to assess monocyte populations.

**Figure S2:** Marker expression in the four different UMAP populations separated according to age. Whole blood was assessed by flow cytometry and monocytes were identified as being Lineage negative HLA‐DR+. Bioinformatic analysis was performed on monocytes and analysed based upon the monocyte markers SLAN, CLA, CCR2, CD14, CD16, CD86, HLA‐DR, and CX3CR1. Marker expression in the four groups identified in Figure 1F are split according to age.

**Figure S3:** No significant difference in cytokine production from monocytes with age. Monocytes were isolated from the peripheral blood and sorted into three populations classical (CD14+), intermediate (CD14 + CD16+) and non‐classical (CD16+), cells were cultured (unstimulated) for 24 h. Cumulative data showing cytokine production from sorted monocyte populations separated according to age.

**Figure S4:** Protein expression within the sorted monocyte populations. Monocytes were isolated from the peripheral blood and sorted into three populations classical (CD14+; red), intermediate (CD14 + CD16+; green) and non‐classical (CD16+; blue), proteomics analysis was performed on the cell pellets. (A) PCA analysis of the sorted monocyte populations and (B) CD14 and CD16 protein expression in the sorted monocyte populations to ensure successful sort and (C) total protein number in the sorted monocyte populations and (D) and PCA analysis separated according to age of donor with young (white) and old (grey).

**Figure S5:** Phagocytosis proteins are significantly downregulated in older monocytes. Phagocytosis associated protein expression classical (CD14+; red), intermediate (CD14 + CD16+; green), and non‐classical (CD16+; blue) in sorted monocyte populations.

**Figure S6:** Increased age in males does not correlate with Complement proteins or decreased monocyte phagocytosis. (A) Serum samples were assessed for C3 and C4 concentrations by ELISA. C3 and C4 serum concentration was correlated in female (pink downwards triangle) and in males (blue upwards triangle). (B) whole blood phagocytosis was performed in young (white) and old (grey) male donors and phagocytosis was assessed by internalisation of BioParticles, data was normalised to the average young CD14+ phagocytosis and assessed in CD14+, CD14 + CD16+ and CD16+ monocytes. (A) Data assessed by Pearson's correlation test.

**Figure S7:** Complement receptor expression on monocytes. Proteomic data set was assessed for complement receptor protein copy number in CD14+, CD14 + CD16+ and CD16+ populations. **p < 0.01; ****p < 0.0001.

**Figure S8:** The frequency of monocyte populations correlated with serum CRP split according to biological sex. Serum C reactive protein (CRP) concentrations were assessed by ELISA and correlated with frequency of monocyte populations in the peripheral blood. Data was split according to whether the donor was female (top) or male (bottom). Assessed by Pearson's correlation test.

**Figure S9:** The route of female sex hormone administration does not impact upon circulating CRP, C3, C4 or monocytes phenotype. Data from Figure 6 was split according to the route of female sex hormone administration. With oestrogen being split according to whether it was received via the cutaneous route or oral route and circulating. The impact of oestrogen administration was assessed for (A) CRP, (B) C3 and C4 and (C) frequency of CD14+, CD14 + CD16+, and CD16+ monocytes. Progesterone was split according to whether it was received via the oral route or locally in the uterus via the coil. The impact of progesterone administration was assessed for (D) CRP, (E) C3 and C4 (F) frequency of CD14+, CD14 + CD16+ and CD16+ monocytes. Data was assessed by a Mann–Whitney test.

**Table S1:** Table of differentially regulated protein found in the heatmap of Figure 3A.

**Table S2:** Table of differentially regulated proteins according to the monocyte cell type.

**Table S3:** Table of age‐associated differentially regulated proteins.

## Data Availability

The data that support the findings of this study are available on request from the corresponding author. The data are not publicly available due to privacy or ethical restrictions.
