# Supplementary figures and images for: Age‐Associated Inflammatory Monocytes Are Increased in Menopausal Females and Reversed by Hormone Replacement Therapy
Source: Aging Cell. 2025 Oct 9;24(11):e70249. doi: 10.1111/acel.70249 (PMC12611317; doi:10.1111/acel.70249)

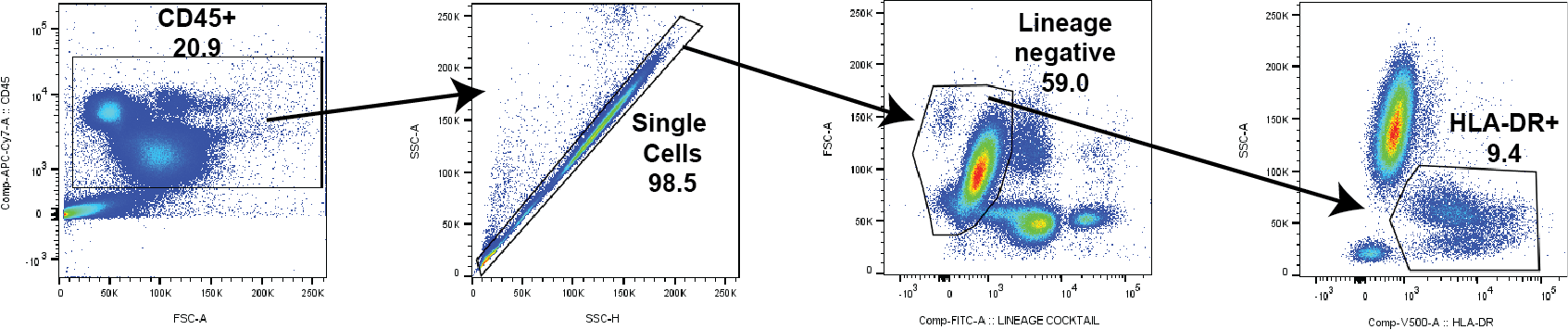

Supplement: Supplementary file 1 — Figure S1: Example gating strategy to identify monocytes in whole blood. Figure shows representative gating strategy to identify monocytes in whole blood that was assessed by flow cytometry. Leukocytes were identified as being CD45+; subsequently, single cells were identified using SSc‐A and SSc‐H. Next lineage negative (CD3, CD19, CD20, and CD56) cells were identified then HLA‐DR+ cells were selected to assess monocyte populations. [file ACEL-24-e70249-s009.png]

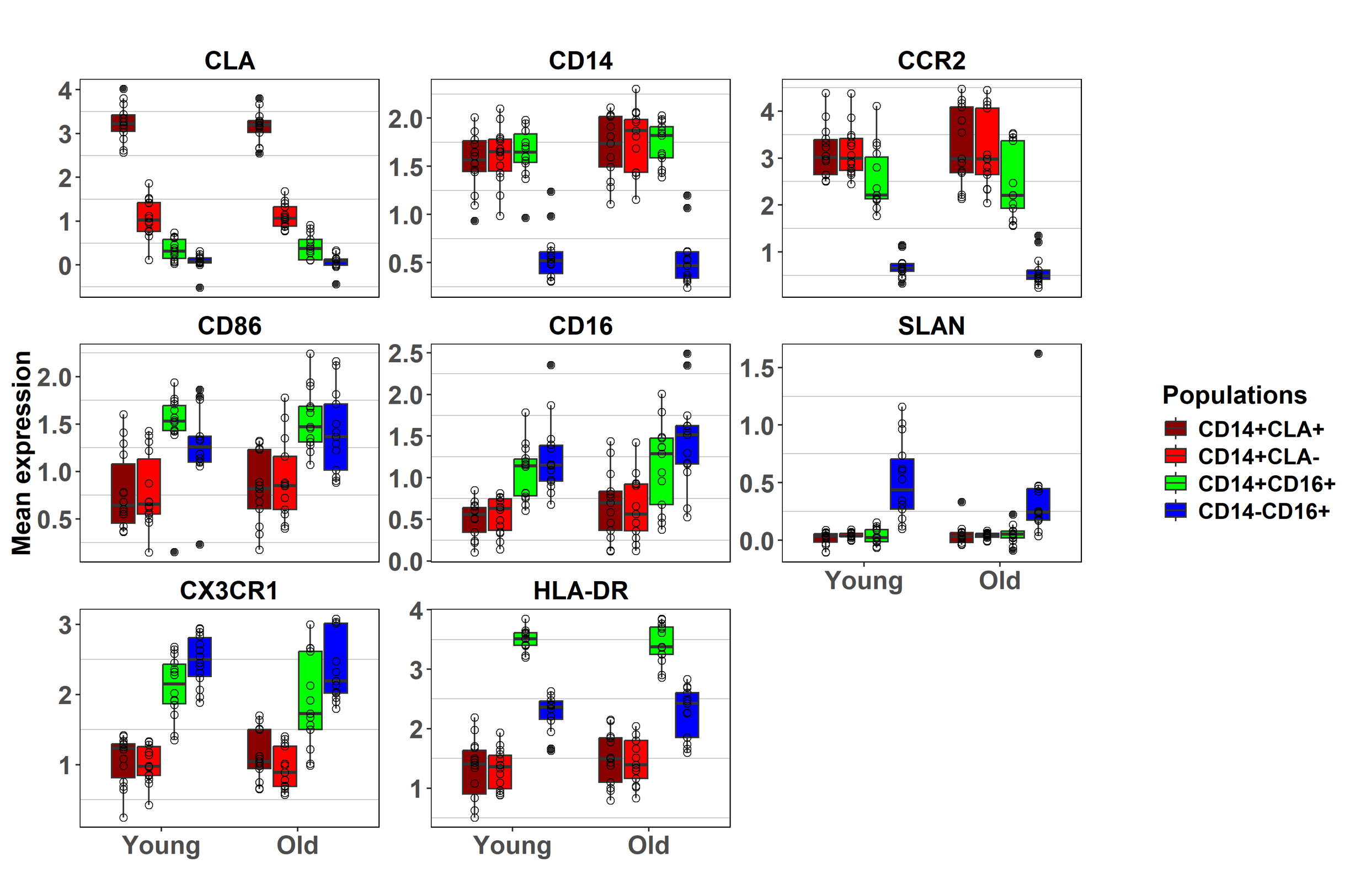

Supplement: Supplementary file 2 — Figure S2: Marker expression in the four different UMAP populations separated according to age. Whole blood was assessed by flow cytometry and monocytes were identified as being Lineage negative HLA‐DR+. Bioinformatic analysis was performed on monocytes and analysed based upon the monocyte markers SLAN, CLA, CCR2, CD14, CD16, CD86, HLA‐DR, and CX3CR1. Marker expression in the four groups identified in Figure 1F are split according to age. [file ACEL-24-e70249-s007.png]

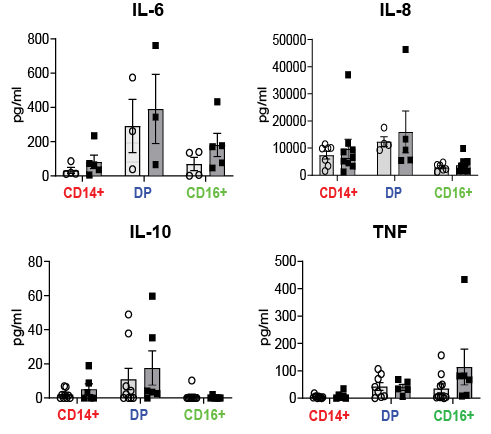

Supplement: Supplementary file 3 — Figure S3: No significant difference in cytokine production from monocytes with age. Monocytes were isolated from the peripheral blood and sorted into three populations classical (CD14+), intermediate (CD14 + CD16+) and non‐classical (CD16+), cells were cultured (unstimulated) for 24 h. Cumulative data showing cytokine production from sorted monocyte populations separated according to age. [file ACEL-24-e70249-s003.png]

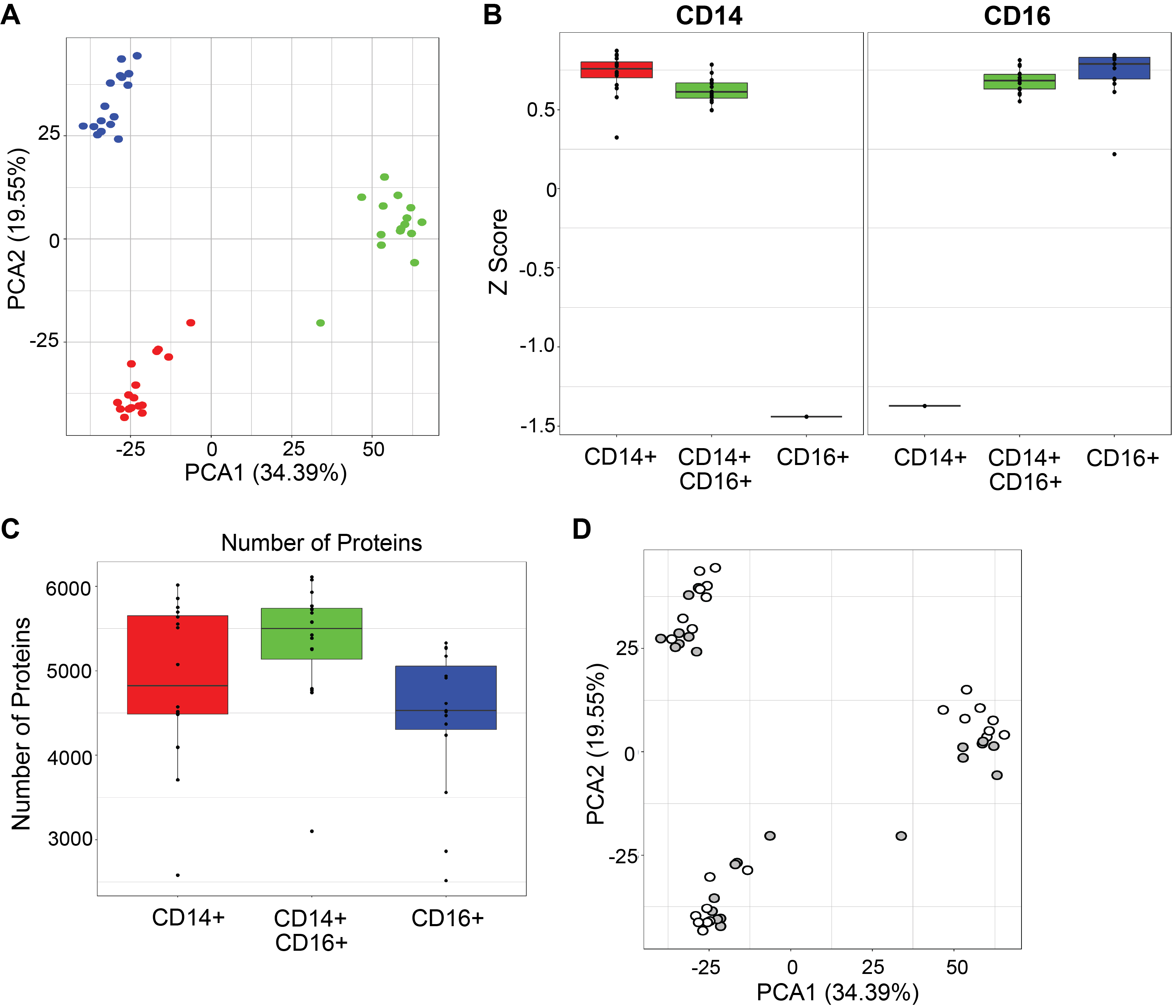

Supplement: Supplementary file 4 — Figure S4: Protein expression within the sorted monocyte populations. Monocytes were isolated from the peripheral blood and sorted into three populations classical (CD14+; red), intermediate (CD14 + CD16+; green) and non‐classical (CD16+; blue), proteomics analysis was performed on the cell pellets. (A) PCA analysis of the sorted monocyte populations and (B) CD14 and CD16 protein expression in the sorted monocyte populations to ensure successful sort and (C) total protein number in the sorted monocyte populations and (D) and PCA analysis separated according to age of donor with young (white) and old (grey). [file ACEL-24-e70249-s004.png]

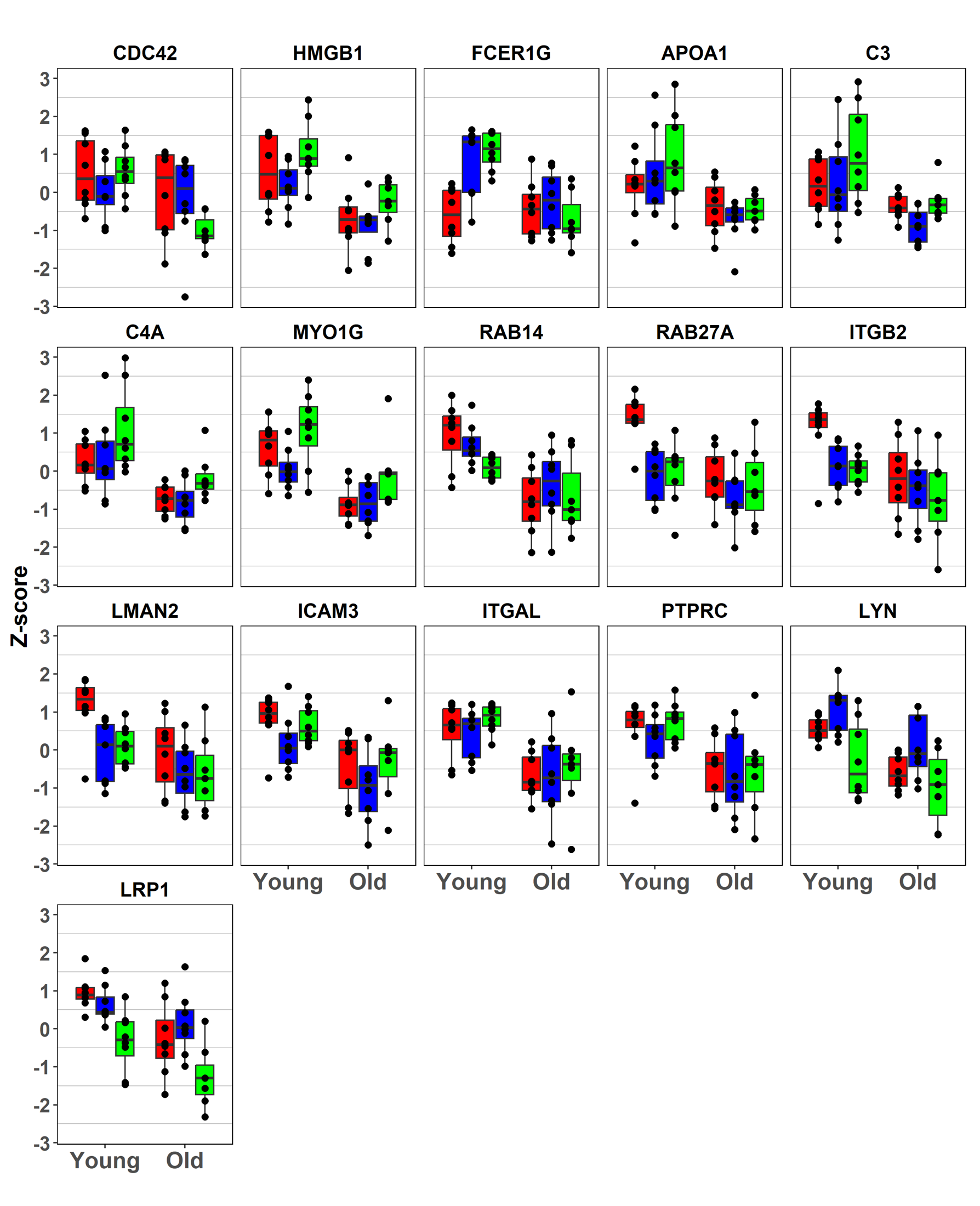

Supplement: Supplementary file 5 — Figure S5: Phagocytosis proteins are significantly downregulated in older monocytes. Phagocytosis associated protein expression classical (CD14+; red), intermediate (CD14 + CD16+; green), and non‐classical (CD16+; blue) in sorted monocyte populations. [file ACEL-24-e70249-s002.png]

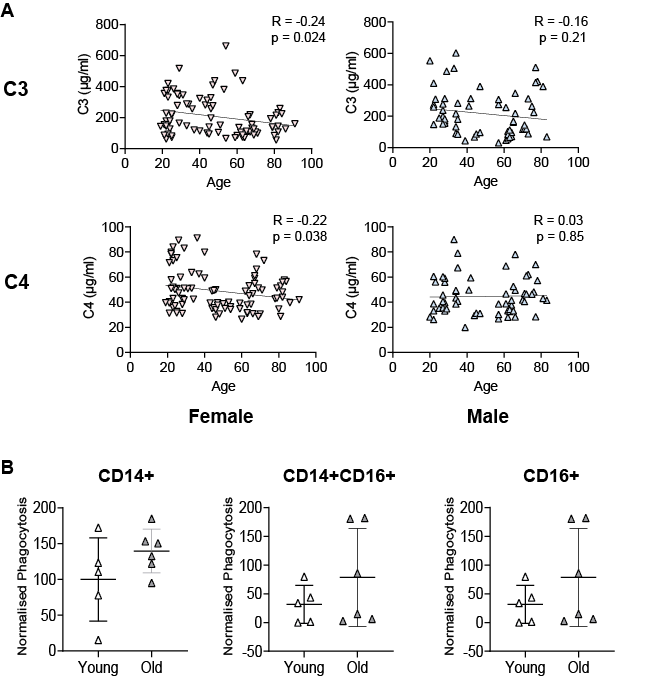

Supplement: Supplementary file 6 — Figure S6: Increased age in males does not correlate with Complement proteins or decreased monocyte phagocytosis. (A) Serum samples were assessed for C3 and C4 concentrations by ELISA. C3 and C4 serum concentration was correlated in female (pink downwards triangle) and in males (blue upwards triangle). (B) whole blood phagocytosis was performed in young (white) and old (grey) male donors and phagocytosis was assessed by internalisation of BioParticles, data was normalised to the average young CD14+ phagocytosis and assessed in CD14+, CD14 + CD16+ and CD16+ monocytes. (A) Data assessed by Pearson's correlation test. [file ACEL-24-e70249-s001.png]

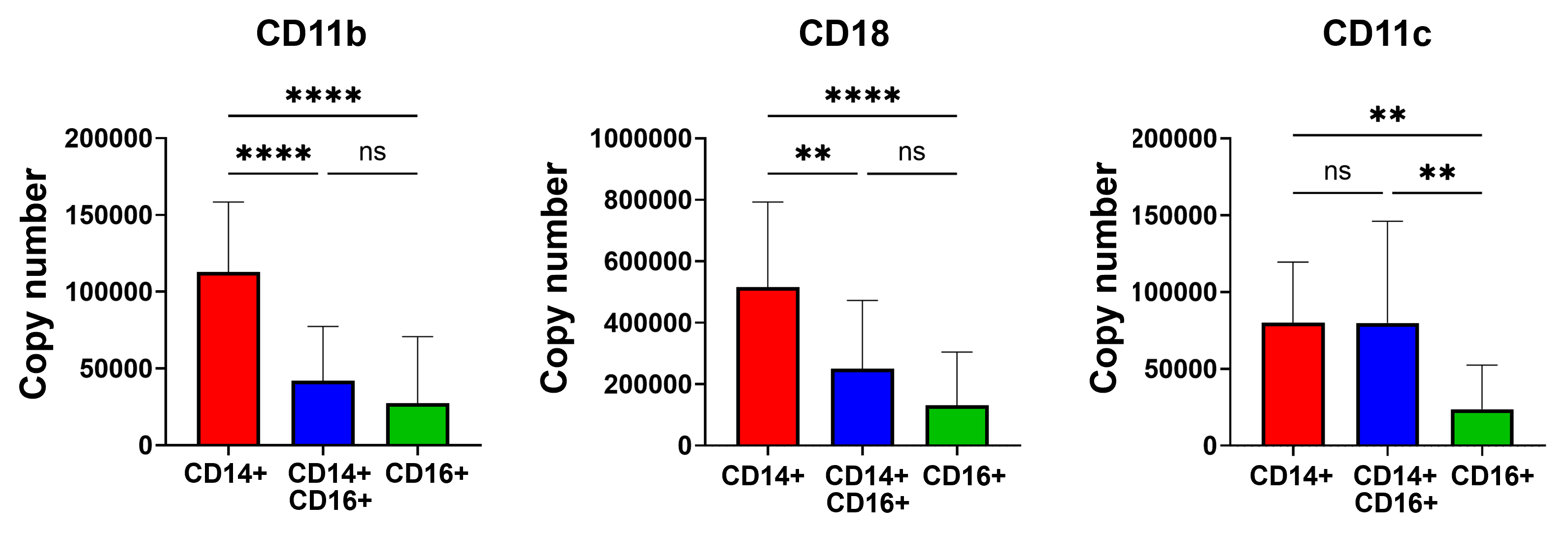

Supplement: Supplementary file 7 — Figure S7: Complement receptor expression on monocytes. Proteomic data set was assessed for complement receptor protein copy number in CD14+, CD14 + CD16+ and CD16+ populations. **p < 0.01; ****p < 0.0001. [file ACEL-24-e70249-s010.png]

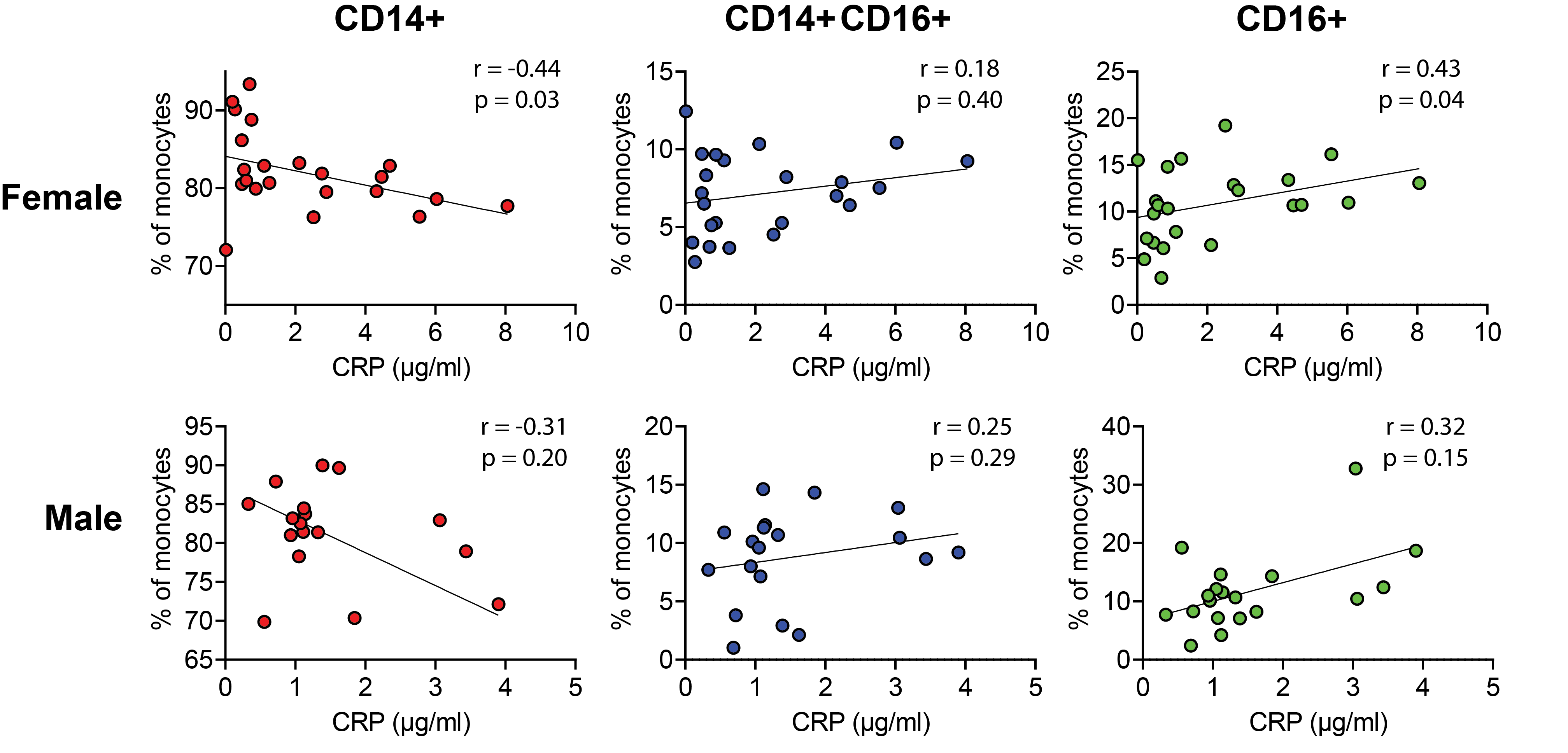

Supplement: Supplementary file 8 — Figure S8: The frequency of monocyte populations correlated with serum CRP split according to biological sex. Serum C reactive protein (CRP) concentrations were assessed by ELISA and correlated with frequency of monocyte populations in the peripheral blood. Data was split according to whether the donor was female (top) or male (bottom). Assessed by Pearson's correlation test. [file ACEL-24-e70249-s012.png]

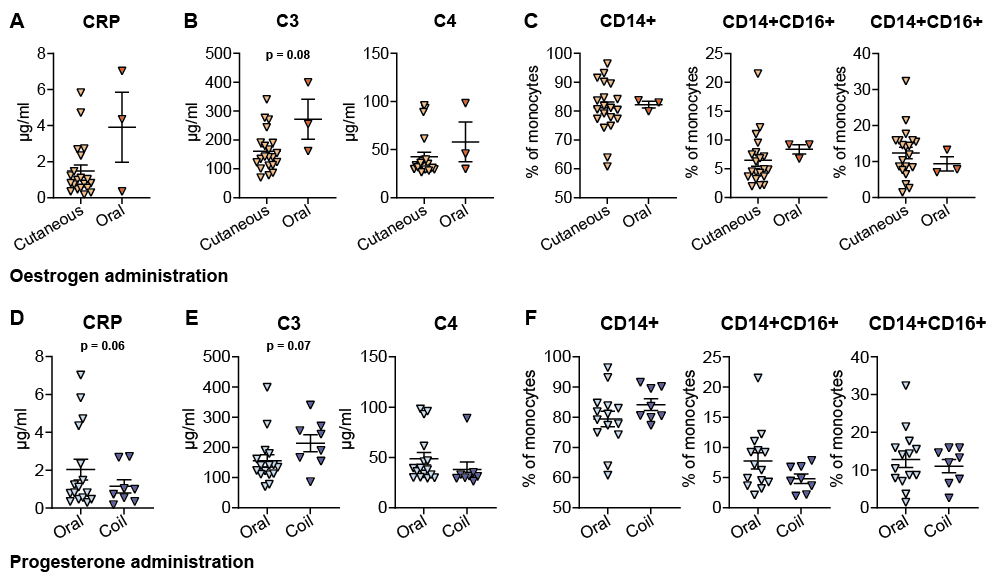

Supplement: Supplementary file 9 — Figure S9: The route of female sex hormone administration does not impact upon circulating CRP, C3, C4 or monocytes phenotype. Data from Figure 6 was split according to the route of female sex hormone administration. With oestrogen being split according to whether it was received via the cutaneous route or oral route and circulating. The impact of oestrogen administration was assessed for (A) CRP, (B) C3 and C4 and (C) frequency of CD14+, CD14 + CD16+, and CD16+ monocytes. Progesterone was split according to whether it was received via the oral route or locally in the uterus via the coil. The impact of progesterone administration was assessed for (D) CRP, (E) C3 and C4 (F) frequency of CD14+, CD14 + CD16+ and CD16+ monocytes. Data was assessed by a Mann–Whitney test. [file ACEL-24-e70249-s008.png]
