# Supplementary material for: Age‐Associated Inflammatory Monocytes Are Increased in Menopausal Females and Reversed by Hormone Replacement Therapy
Source: Aging Cell. 2025 Oct 9;24(11):e70249. doi: 10.1111/acel.70249 (PMC12611317; doi:10.1111/acel.70249)
Supplement: Supplementary file 10 — Table S1: Table of differentially regulated protein found in the heatmap of Figure 3A. [file ACEL-24-e70249-s005.pdf]

Heatmap order	Protein Accessions	Gene Names	Protein Descriptions
1	P32455	GBP1	Guanylate-binding protein 1
2	P12277	CKB	Creatine kinase B-type
3	P43490	NAMPT	Nicotinamide phosphoribosyltransferase
4	P04179	SOD2	Superoxide dismutase [Mn], mitochondrial
5	Q9BW30	TPPP3	Tubulin polymerization-promoting protein family member 3
6	Q08257	CRYZ	Quinone oxidoreductase
7	Q8IV04	TBC1D10C	Carabin
8	Q9HC16	APOBEC3G	DNA dC->dU-editing enzyme APOBEC-3G
9	O14796	SH2D1B	SH2 domain-containing protein 1B
10	P51608-2	MECP2	Isoform B of Methyl-CpG-binding protein 2
11	Q86YV0	RASAL3	RAS protein activator like-3
12	P53634	CTSC	Dipeptidyl peptidase 1
13	P14222	PRF1	Perforin-1
14	P42330	AKR1C3	Aldo-keto reductase family 1 member C3
15	O76096	CST7	Cystatin-F
16	P59768	GNG2	Guanine nucleotide-binding protein G(I)/G(S)/G(O) subunit gamma-2
17	Q7Z7L1	SLFN11	Schlafen family member 11
18	Q7Z4H3	HDDC2	5'-deoxynucleotidase HDDC2
19	O75791	GRAP2	GRB2-related adapter protein 2
20	Q08AF3	SLFN5	Schlafen family member 5
21	Q9BPX5	ARPC5L	Actin-related protein 2/3 complex subunit 5-like protein
22	Q14847	LASP1	LIM and SH3 domain protein 1
23	Q9UI08-2	EVL	Isoform 1 of Ena/VASP-like protein
24	Q9BUH6	PAXX	C16
25	O43670;O43670-2;O43670-4	ZNF207	BUB3-interacting and GLEBS motif-containing protein ZNF207;Isoform 2 of BUB3-interacting and GLEBS motif-containing protein ZNF207;Isoform 4 of BUB3-interacting and GLEBS motif-containing protein ZNF207
26	P33240;P33240-2	CSTF2	Cleavage stimulation factor subunit 2;Isoform 2 of Cleavage stimulation factor subunit 2
27	P00813	ADA	Adenosine deaminase
28	O95865	DDAH2	N(G),N(G)-dimethylarginine dimethylaminohydrolase 2
29	Q15942	ZYX	Zyxin

30	Q05315	CLC	Galectin-10
31	P63261	ACTG1	Actin, cytoplasmic 2
32	Q05519;Q05519-2	SRSF11	Serine/arginine-rich splicing factor 11;Isoform 2 of Serine/arginine-rich splicing factor 11
33	P01834	IGKC	Immunoglobulin kappa constant
34	P0CG38	POTEI	POTE ankyrin domain family member I
35	P68871	HBB	Hemoglobin subunit beta
36	O43516;O43516-3	WIPF1	WAS/WASL-interacting protein family member 1;Isoform 3 of WAS/WASL-interacting protein family member 1
37	P08134	RHOC	Rho-related GTP-binding protein RhoC
38	Q9P2A4;Q9P2A4-2	ABI3	ABI gene family member 3;Isoform 2 of ABI gene family member 3
39	O00479	HMGN4	High mobility group nucleosome-binding domain-containing protein 4
40	P16402	H1-3	Histone H1.3
41	P22304	IDS	Iduronate 2-sulfatase
42	Q8NFU3	TSTD1	Thiosulfate:glutathione sulfurtransferase
43	Q9Y6X5	ENPP4	Bis(5'-adenosyl)-triphosphatase ENPP4
44	P02452	COL1A1	Collagen alpha-1(I) chain
45	Q04756	HGFAC	Hepatocyte growth factor activator
46	Q8WWZ4;Q8WWZ4-2	ABCA10	ATP-binding cassette sub-family A member 10;Isoform 2 of ATP-binding cassette sub-family A member 10
47	P36955	SERPINF1	Pigment epithelium-derived factor
48	P51884	LUM	Lumican
49	Q96RW7;Q96RW7-2	HMCN1	Hemicentin-1;Isoform 2 of Hemicentin-1
50	Q8IVL0;Q8IVL0-2;Q8IVL0-3	NAV3	Neuron navigator 3;Isoform 2 of Neuron navigator 3;Isoform 3 of Neuron navigator 3
51	P12109	COL6A1	Collagen alpha-1(VI) chain
52	Q9NYH9	UTP6	U3 small nucleolar RNA-associated protein 6 homolog
53	O95498;O95498-6	VNN2	Pantetheine hydrolase VNN2;Isoform 6 of Pantetheine hydrolase VNN2
54	P04004	VTN	Vitronectin

55	P05543	SERPINA7	Thyroxine-binding globulin
56	P16403	H1-2	Histone H1.2
57	Q8IUE6	H2AC21	Histone H2A type 2-B
58	Q9BTD8;Q9BTD8-2;Q9BTD8-3;Q9BTD8-4	RBM42	RNA-binding protein 42;Isoform 2 of RNA-binding protein 42;Isoform 3 of RNA-binding protein 42;Isoform 4 of RNA-binding protein 42
59	O00151	PDLIM1	PDZ and LIM domain protein 1
60	P18065	IGFBP2	Insulin-like growth factor-binding protein 2
61	Q8IWC1;Q8IWC1-3;Q8IWC1-4	MAP7D3	MAP7 domain-containing protein 3;Isoform 3 of MAP7 domain-containing protein 3;Isoform 4 of MAP7 domain-containing protein 3
62	P69905	HBA1	Hemoglobin subunit alpha
63	Q14118	DAG1	Dystroglycan 1
64	P18859;P18859-2	ATP5PF	ATP synthase-coupling factor 6, mitochondrial;Isoform 2 of ATP synthase-coupling factor 6, mitochondrial
65	P02649	APOE	Apolipoprotein E
66	P02656	APOC3	Apolipoprotein C-III
67	P17927	CR1	Complement receptor type 1
68	P13611	VCAN	Versican core protein
69	O95347;O95347-2	SMC2	Structural mainte0ce of chromosomes protein 2;Isoform 2 of Structural mainte0ce of chromosomes protein 2
70	P25205	MCM3	DNA replication licensing factor MCM3
71	P02792	FTL	Ferritin light chain
72	P09601	HMOX1	Heme oxygenase 1
73	P31941	APOBEC3A	DNA dC->dU-editing enzyme APOBEC-3A
74	P49419;P49419-2	ALDH7A1	Alpha-aminoadipic semialdehyde dehydrogenase;Isoform 2 of Alpha-aminoadipic semialdehyde dehydrogenase
75	P09668	CTSH	Pro-cathepsin H
76	P06737;P06737-2	PYGL	Glycogen phosphorylase, liver form;Isoform 2 of Glycogen phosphorylase, liver form
77	P08758	ANXA5	Annexin A5
78	P50225	SULT1A1	Sulfotransferase 1A1
79	Q9NZK5	ADA2	Adenosine deaminase 2

80	O94819	KBTBD11	Kelch repeat and BTB domain-containing protein 11
81	P50452	SERPINB8	Serpin B8
82	P50135	HNMT	Histamine N-methyltransferase
83	Q8TEH3;Q8TEH3-2;Q8TEH3-3	DENND1A	DENN domain-containing protein 1A;Isoform 2 of DENN domain-containing protein 1A;Isoform 3 of DENN domain-containing protein 1A
84	Q7L266	ASRGL1	Isoaspartyl peptidase/L-asparaginase
85	P52209;P52209-2	PGD	6-phosphogluconate dehydrogenase, decarboxylating;Isoform 2 of 6-phosphogluconate dehydrogenase, decarboxylating
86	Q12882	DPYD	Dihydropyrimidine dehydrogenase [NADP(+)]
87	O75874	IDH1	Isocitrate dehydrogenase [NADP] cytoplasmic
88	P19878	NCF2	Neutrophil cytosol factor 2
89	P40121	CAPG	Macrophage-capping protein
90	O95716	RAB3D	Ras-related protein Rab-3D
91	Q02338	BDH1	D-beta-hydroxybutyrate dehydrogenase, mitochondrial
92	P00738	HP	Haptoglobin
93	P08631;P08631-4	HCK	Tyrosine-protein kinase HCK;Isoform 4 of Tyrosine-protein kinase HCK
94	Q9H3G5	CPVL	Probable serine carboxypeptidase CPVL
95	P05091	ALDH2	Aldehyde dehydrogenase, mitochondrial
96	Q96CX2	KCTD12	BTB/POZ domain-containing protein KCTD12
97	P30043	BLVRB	Flavin reductase (NADPH)
98	P41218	MNDA	Myeloid cell nuclear differentiation antigen
99	Q9UBR2	CTSZ	Cathepsin Z
100	P07858	CTSB	Cathepsin B
101	P23141;P23141-2	CES1	Liver carboxylesterase 1;Isoform 2 of Liver carboxylesterase 1
102	Q03169	TNFAIP2	Tumor necrosis factor alpha-induced protein 2

103	Q12797;Q12797-10	ASPH	Aspartyl/asparaginyl beta-hydroxylase;Isoform 10 of Aspartyl/asparaginyl beta-hydroxylase
104	P28676	GCA	Grancalcin
105	P05109	S100A8	Protein S100-A8
106	P06702	S100A9	Protein S100-A9
107	O00602	FCN1	Ficolin-1
108	P01903	HLA-DRA	HLA class II histocompatibility antigen, DR alpha chain
109	Q04941	PLP2	Proteolipid protein 2
110	Q9Y336	SIGLEC9	Sialic acid-binding Ig-like lectin 9
111	P10620	MGST1	Microsomal glutathione S-transferase 1
112	Q07065	CKAP4	Cytoskeleton-associated protein 4
113	Q8WY22	BRI3BP	BRI3-binding protein
114	O94905	ERLIN2	Erlin-2
115	Q8NBQ5	HSD17B11	Estradiol 17-beta-dehydrogenase 11
116	Q96D96	HVCN1	Voltage-gated hydrogen channel 1
117	Q9H8H3	METTL7A	Putative methyltransferase-like protein 7A
118	A6NI72	NCF1B	Putative neutrophil cytosol factor 1B
119	O43175	PHGDH	D-3-phosphoglycerate dehydrogenase
120	Q15080	NCF4	Neutrophil cytosol factor 4
121	Q6P4A8	PLBD1	Phospholipase B-like 1
122	Q13637	RAB32	Ras-related protein Rab-32
123	P61626	LYZ	Lysozyme C
124	P05164;P05164-2;P05164-3	MPO	Myeloperoxidase;Isoform H14 of Myeloperoxidase;Isoform H7 of Myeloperoxidase
125	P08311	CTSG	Cathepsin G
126	P24158	PRTN3	Myeloblastin
127	P08571	CD14	Monocyte differentiation antigen CD14
128	O95197-3	RTN3	Isoform 3 of Reticulon-3
129	Q9NUU6	OTULINL	Inactive ubiquitin thioesterase OTULINL
130	P04839	CYBB	Cytochrome b-245 heavy chain
131	P13498	CYBA	Cytochrome b-245 light chain
132	P10153	RNASE2	Non-secretory ribonuclease
133	Q9UM07	PADI4	Protein-arginine deiminase type-4
134	P80723	BASP1	Brain acid soluble protein 1
135	P08246	ELANE	Neutrophil elastase
136	P20160	AZU1	Azurocidin
