# Supplementary material for: Age‐Associated Inflammatory Monocytes Are Increased in Menopausal Females and Reversed by Hormone Replacement Therapy
Source: Aging Cell. 2025 Oct 9;24(11):e70249. doi: 10.1111/acel.70249 (PMC12611317; doi:10.1111/acel.70249)
Supplement: Supplementary file 11 — Table S2: Table of differentially regulated proteins according to the monocyte cell type. [file ACEL-24-e70249-s006.pdf]

		CD14+ vs CD14+CD16+			CD14+CD16+ vs CD16+			CD14+ vs CD16+		
**Protein_id**	**Gene name**	**log2fold change**	**p value**	**qvalue**	**log2fold change**	**p value**	**qvalue**	**log2fold change**	**p value**	**qvalue**
P51608-2	MECP2	0.435727671	0.020489763	0.067019393	2.391711137	2.63E-12	4.15E-10	2.555172753	1.15E-10	7.86E-09
O43583	DENR	0.387178923	0.008085557	0.031712651	0.580922159	9.13E-05	0.000820871	0.986418715	6.37E-06	6.84E-05
Q9UJ70	NAGK	0.672555291	5.49E-05	0.000509383	-1.326752227	5.88E-05	0.000580728	-0.804019133	0.011065955	0.033982685
P06744	GPI	-1.94E-15	0.999999916	1	-0.624637451	0.011027536	0.038748383	-1.068007999	4.71E-05	0.000366531
P27708	CAD	-0.226606749	0.032458249	0.097236861	-0.505999043	0.01374779	0.045891431	-0.673381755	0.005537606	0.019156724
Q9NXG2	THUMPD1	0.29308227	0.07103011	0.181487078	0.934417243	3.68E-07	9.06E-06	0.924969895	0.000106082	0.000732809
Q16831	UPP1	0.341729932	0.042285035	0.120142654	0.523434145	0.001546992	0.008084015	0.613960298	0.003256872	0.012192152
Q96C86	DCPS	0.591569826	2.93E-06	3.88E-05	0.745823311	1.23E-06	2.52E-05	1.096322378	7.11E-09	2.37E-07
P09467	FBP1	0.430047513	0.000895877	0.005186969	-1.158212147	1.77E-05	0.000215295	-0.776059673	0.003095051	0.01170443
P04040	CAT	0.352384584	0.010880243	0.040171451	-0.914551417	6.84E-05	0.000645829	-0.73812288	0.000927348	0.004380455
Q9Y4E8	USP15	0.008028629	0.722008216	1	-0.749985256	1.57E-05	0.000196614	-1.18890531	1.32E-11	1.50E-09
P09874	PARP1	-0.102611363	0.248245547	0.483598784	1.061053491	1.28E-19	1.19E-16	0.509426554	0.000219738	0.001303377
Q01081	U2AF1	0.640440034	0.008822163	0.034108495	0.616330729	0.004481371	0.019010235	1.053824334	0.000160282	0.001010709
Q13637	RAB32	-4.32E-17	0.999999991	1	-1.658278102	7.36E-07	1.60E-05	-1.391572288	1.69E-05	0.000153134
P33240;P33240-2	CSTF2	0.755375511	0.002071839	0.010285411	1.042953362	9.77E-08	2.88E-06	1.547977527	7.71E-09	2.54E-07
P06737;P06737-2	PYGL	-0.023589787	0.600456673	0.993528967	-1.255755273	0.000188202	0.001453767	-1.823494595	3.41E-07	5.87E-06
Q8WVY7	UBLCP1	0.203356062	0.05520747	0.149739645	0.973866692	5.04E-10	2.88E-08	1.014350964	4.22E-08	1.10E-06
Q00610;Q00610-2	CLTC	6.32E-16	0.999999962	1	-0.634903933	4.28E-06	7.01E-05	-0.684112019	0.000830751	0.003989447
P60981	DSTN	0.502804636	0.000234792	0.001686104	0.87949583	2.68E-09	1.17E-07	1.119672477	5.42E-08	1.34E-06
Q96C19	EFHD2	0.749321087	3.71E-11	2.18E-09	0.890444863	3.55E-10	2.21E-08	1.330327582	2.04E-15	6.61E-13
P82979	SARNP	1.627032895	7.61E-05	0.000662593	0.826936222	0.008142475	0.031063265	2.010016241	5.96E-05	0.000449318
P00338	LDHA	0.142611606	0.151962521	0.330345159	-0.591013604	0.000431951	0.002883786	-0.521297882	0.000573844	0.002958031
P48426	PIP4K2A	0.482118558	6.85E-05	0.000614396	1.134866151	2.67E-12	4.15E-10	1.364548976	6.27E-15	1.63E-12
Q9UI08-2	EVL	2.141250278	5.59E-43	1.97E-39	1.348727333	1.45E-10	1.10E-08	3.085532738	4.06E-18	2.63E-15
Q01469	FABP5	-0.368343685	0.001794596	0.009085442	-0.566614693	0.012164551	0.041749572	-1.167461534	7.38E-06	7.67E-05
Q04917	YWHAH	0.059031243	0.50936262	0.86471478	-0.815972248	1.36E-10	1.06E-08	-0.646797692	3.98E-05	0.000318753
P62258	YWHAE	0.257827774	0.003632789	0.016401042	-0.835234932	1.01E-11	1.18E-09	-0.780561257	1.50E-09	6.30E-08
P30740	SERPINB1	-0.275462857	0.075547317	0.190188187	-0.722659074	0.000631117	0.003923843	-1.372099597	1.88E-07	3.70E-06
P37837	TALDO1	0.31548715	0.005469346	0.022964643	-0.75946021	0.000856722	0.004973597	-0.596281688	0.009592397	0.030134279
P50452	SERPINB8	-0.264473793	0.04349557	0.123284068	-1.155875551	1.47E-06	2.89E-05	-1.697218695	3.00E-10	1.69E-08
P52790	HK3	0.506635511	0.000265741	0.001864433	-1.384951838	0.001245718	0.006717292	-1.395984866	0.001881996	0.007785711
P50225	SULT1A1	0.295501412	0.150812313	0.328958916	-1.497369978	0.000116062	0.000977288	-1.37013428	0.000445293	0.002375507
O14745	SLC9A3R1	0.225144132	0.085466815	0.209745203	1.610396745	1.98E-20	5.56E-17	1.688846388	1.96E-13	3.63E-11
Q8ND71	GIMAP8	0.386295457	0.00108799	0.00605087	-0.74215579	2.71E-05	0.000308923	-0.576362398	2.03E-05	0.000180916
P10412	H1-4	0.469749872	0.134411397	0.301454077	1.010167322	0.011882611	0.041033057	1.523631918	0.000994306	0.004671259
P16402	H1-3	1.523814224	0.002016493	0.010042593	1.536276752	0.010119937	0.036473397	3.020124095	1.99E-05	0.000178402
Q15102	PAFAH1B3	0	1	1	-0.710991871	0.000395779	0.002726693	-1.089264247	1.18E-09	5.20E-08
Q07955	SRSF1	0.110192854	0.430318047	0.753729701	0.511887183	0.005664751	0.023087154	0.603918685	0.005838627	0.019880412
P19878	NCF2	0.068165491	0.493743436	0.841846884	-2.967819528	3.60E-08	1.17E-06	-3.797735995	1.39E-12	2.06E-10
O43768;O43768-2	ENSA	0.889391406	0.004125587	0.018160824	0.581794324	0.011395173	0.039692008	1.174176143	0.000169044	0.00105318
P06702	S100A9	0.255410186	0.145305418	0.319020488	-3.178246486	1.21E-13	3.09E-11	-3.022987782	2.46E-10	1.45E-08
P21283	ATP6V1C1	5.79E-16	0.99999997	1	-0.975846615	3.76E-05	0.000404395	-1.094416446	5.29E-06	6.00E-05

Q15149	PLEC	0.346935344	0.011287393	0.041457655	-1.240156821	5.78E-12	7.37E-10	-1.00534381	4.48E-07	7.51E-06
Q96D96	HVCN1	-6.43E-17	0.999999995	1	-1.596488622	4.05E-05	0.000421771	-1.774383728	3.89E-05	0.000314848
Q9NVA2	SEPTIN11	7.36E-16	0.999999947	1	0.754940417	1.28E-07	3.56E-06	0.566521452	3.96E-05	0.000318326
P02768	ALB	0	1	1	0.847589698	0.004823736	0.02021787	0.873817268	0.00232879	0.009222859
P51452	DUSP3	0.428997223	0.003403665	0.015525644	-1.183000878	0.000231916	0.001762313	-0.996334553	0.001233169	0.005542862
Q14554	PDIA5	-0.143446721	0.232711725	0.460978394	-0.848107252	0.006429833	0.025598295	-0.939324528	0.003172134	0.011926489
P35475	IDUA	0.253511209	0.050895584	0.140091981	0.998681235	3.74E-05	0.000402954	1.126569221	3.50E-06	4.21E-05
P11586	MTHFD1	-1.07E-17	0.999999998	1	-0.528989091	0.000104805	0.000912646	-0.836602664	1.03E-08	3.28E-07
Q709C8;Q709C8-3	VPS13C	1.41E-15	0.99999992	1	-0.53155076	7.50E-06	0.000110739	-0.705167967	8.56E-07	1.32E-05
P08311	CTSG	-0.545406184	0.084347381	0.207978227	-3.012987709	5.33E-07	1.27E-05	-4.637119632	1.23E-09	5.33E-08
P05091	ALDH2	0.278342413	0.034666495	0.102459397	-2.41303816	9.65E-09	3.51E-07	-2.350301708	1.33E-08	4.10E-07
Q7L1T6	CYB5R4	0.028059521	0.664716254	1	1.148498682	0.004203135	0.018103826	1.159059099	0.002961904	0.01128303
Q7Z3J2	VPS35L	1.31E-16	0.999999983	1	-0.677264161	0.000319912	0.002270969	-1.040685952	2.48E-06	3.17E-05
P09769	FGR	0.251706825	0.014702282	0.051023864	-1.120585233	2.64E-09	1.17E-07	-1.079305734	1.39E-09	5.92E-08
P08631;P08631-4	HCK	0.709938131	1.00E-08	2.80E-07	-1.465575653	1.48E-05	0.000188752	-1.174799633	1.81E-05	0.000162452
Q9P289	STK26	0.430149581	9.60E-05	0.000809666	0.757160869	8.03E-13	1.61E-10	1.030424582	1.39E-10	9.24E-09
P31146	CORO1A	0.841101049	1.48E-11	9.86E-10	0.657100779	4.82E-06	7.68E-05	1.226363405	7.13E-11	5.73E-09
Q9HAF1;Q9HAF1-2;Q9HAF1-3;Q9HAF1-4	MEAF6	0.590569125	0.028221337	0.08690693	0.818065105	0.000264074	0.001943476	1.058107339	0.005691498	0.019517619
P0DOY3	IGLC3	1.035965966	3.18E-08	7.38E-07	0.631558258	0.000555398	0.003531376	1.381707435	2.11E-08	5.90E-07
P09104	ENO2	3.61E-17	0.999999997	1	-0.49892957	0.010574446	0.037627851	-1.376658086	2.81E-07	5.12E-06
P06733	ENO1	0.437127083	0.001431928	0.007502196	-1.063126365	1.68E-07	4.57E-06	-0.887837485	6.60E-05	0.000488774
Q96QK1	VPS35	6.49E-17	0.999999991	1	-0.520592769	0.013045538	0.043965971	-0.798957476	0.000141904	0.000926301
Q99536	VAT1	5.66E-18	0.999999997	1	-0.560327137	9.91E-05	0.000868353	-0.54028067	0.001080573	0.005032592
O43707	ACTN4	0.134110234	0.134483058	0.301454077	0.71996765	3.82E-09	1.58E-07	0.717582493	2.46E-06	3.16E-05
O94973	AP2A2	1.50E-13	0.999999126	1	-0.469018159	0.001147869	0.006298676	-0.650308805	1.37E-06	1.96E-05
Q14141;Q14141-2;Q14141-4	SEPTIN6	0.031735669	0.605760843	1	0.910257795	6.03E-08	1.88E-06	0.781718314	3.32E-06	4.01E-05
P00441	SOD1	0.328734096	0.029906044	0.090825762	0.605449065	2.26E-06	4.16E-05	0.751359721	2.13E-05	0.000187612
P51688	SGSH	1.50E-16	0.99999998	1	1.396486038	3.84E-07	9.28E-06	1.268627728	9.22E-07	1.41E-05
O75165	DNAJC13	-1.53E-14	0.999999743	1	-1.401918393	4.00E-05	0.000421771	-1.634141187	4.35E-06	5.02E-05
Q9NZN3	EHD3	0	1	1	0.614641997	9.09E-06	0.000126772	0.858249722	1.81E-08	5.39E-07
Q9UHD8-5	SEPTIN9	0.620346527	8.51E-07	1.33E-05	0.483029844	0.000890769	0.005128783	0.831861364	6.41E-07	1.03E-05
Q12882	DPYD	4.11E-16	0.999999978	1	-1.493168927	3.60E-05	0.000389241	-1.476985092	1.71E-05	0.000154594
P29966	MARCKS	1.021514738	3.72E-06	4.76E-05	-1.631924613	4.91E-06	7.77E-05	-1.03427584	0.003203745	0.012027934
P49662;P49662-2	CASP4	0.043242384	0.518976918	0.877235194	0.804578189	0.000952166	0.005404601	0.560277827	0.005575008	0.019260465
Q05655	PRKCD	0	1	1	-0.825295589	1.89E-05	0.000225745	-1.2466726	4.77E-10	2.48E-08
P14618	PKM	0.445201805	0.002439196	0.011781652	-1.398214101	3.23E-11	2.92E-09	-1.051113876	9.63E-06	9.55E-05
Q9Y5Z4	HEBP2	-0.122673665	0.276770419	0.530088265	-0.785815667	0.000669003	0.004087081	-1.380407456	7.17E-08	1.68E-06
P46459	NSF	0.191474347	0.022173898	0.071467244	-0.528270249	6.61E-05	0.00063052	-0.604101677	2.61E-06	3.29E-05
Q14166	TTLL12	0.266968758	0.006246263	0.025729351	-0.601038183	0.000437656	0.00290803	-0.572259971	0.002380922	0.009414971
Q6NVY1	HIBCH	0.159309471	0.266749817	0.513125943	0.533724313	7.19E-05	0.000667948	0.601131448	0.008807371	0.028144589
Q684P5;Q684P5-3	RAP1GAP2	0.631421817	3.27E-08	7.53E-07	0.890807002	8.40E-14	2.62E-11	1.266179229	5.05E-16	1.88E-13

Q9H3G5	CPVL	0.927844774	2.35E-08	5.57E-07	-3.002957926	1.42E-13	3.33E-11	-2.166560635	1.83E-08	5.41E-07
Q9HB90	RRAGC	-5.27E-13	0.999998887	1	-0.609856222	0.000266332	0.001949856	-1.19541584	6.89E-10	3.25E-08
O95445	APOM	0	1	1	1.233556496	0.011461507	0.039873529	0.964405767	0.014139063	0.041319781
Q8NBQ5	HSD17B11	0	1	1	-1.174852857	0.000535876	0.003430588	-1.699662526	0.000339265	0.001889281
O43813	LANCL1	0	1	1	0.765480072	4.45E-06	7.13E-05	0.509858042	0.000348477	0.00192365
O00115;O00115-2	DNASE2	0.992766722	2.94E-07	5.13E-06	1.232995105	9.78E-12	1.18E-09	2.152687289	3.56E-11	3.31E-09
O15067	PFAS	5.16E-23	1	1	-0.625769865	0.004164	0.017990533	-0.813613146	0.000107213	0.000736877
O00442	RTCA	0.586262182	0.000164978	0.001243684	0.510463598	0.004234632	0.018183627	0.740527681	0.000215701	0.001290741
Q96P48	ARAP1	-3.18E-18	0.999999999	1	-0.591746341	0.003256028	0.01486955	-0.955442106	6.91E-06	7.24E-05
Q08257	CRYZ	1.09E-17	0.999999996	1	1.499558606	2.39E-09	1.12E-07	1.341750404	1.24E-06	1.84E-05
Q9UBW5	BIN2	0.901376021	1.70E-13	1.66E-11	0.862132447	5.87E-06	8.94E-05	1.288474334	1.00E-16	5.21E-14
O43760;O43760-2	SYNGR2	2.10E-17	0.999999994	1	-1.029885329	1.62E-05	0.000201517	-1.186850553	1.28E-05	0.00012108
Q9HB40	SCPEP1	7.25E-16	0.99999995	1	-0.822760876	0.000723628	0.004307969	-1.037147859	3.31E-05	0.000274381
P32455	GBP1	1.983097415	7.99E-15	1.17E-12	-0.622156953	0.001667786	0.008588154	1.000228002	0.000654708	0.003290003
P0C0S8;Q96KK5;Q99878	H2AC11;H2AC12;H2AC14	0.722977983	0.030810833	0.093027943	1.07981191	0.004551379	0.019238836	1.728857557	1.60E-05	0.00014661
P52209;P52209-2	PGD	-0.084486563	0.421557397	0.74320569	-1.342539008	1.21E-05	0.000163468	-1.902117147	2.73E-09	1.06E-07
Q99439	CNN2	1.048504208	4.37E-06	5.53E-05	0.727374705	0.00021929	0.001680029	1.616074577	6.38E-08	1.51E-06
P30038	ALDH4A1	-0.025608075	0.635902974	1	-0.82056582	0.000660561	0.004052982	-1.443645357	8.11E-08	1.86E-06
P23141;P23141-2	CES1	1.10E-17	0.999999999	1	-2.905007342	2.47E-10	1.76E-08	-2.866395068	1.80E-08	5.39E-07
Q92947	GCDH	0.654626175	0.000799635	0.004746657	0.623218559	2.41E-05	0.000280455	0.954111909	3.00E-05	0.000252233
P21964;P21964-2	COMT	-1.06E-17	0.999999998	1	-0.807523397	0.000511146	0.003325415	-0.864359738	0.003351574	0.012462275
P13489	RNH1	4.21E-16	0.999999964	1	-1.085815756	1.35E-06	2.72E-05	-1.338731843	4.66E-09	1.61E-07
P24666	ACP1	0.747708698	7.65E-06	9.01E-05	0.634528902	5.48E-06	8.53E-05	1.118345441	2.25E-09	8.93E-08
P62979	RPS27A	0.12458085	0.316276698	0.586336298	0.51965441	0.010072788	0.036350192	0.657538799	6.49E-05	0.000483078
Q9UBE0	SAE1	0.269383042	0.009444104	0.036233126	0.556871828	3.20E-05	0.000351498	0.649812347	3.15E-05	0.000263981
P54802	NAGLU	0	1	1	1.163171056	1.33E-06	2.70E-05	0.705633281	0.001809016	0.007540356
P07237	P4HB	2.91E-16	0.999999968	1	-0.603659154	1.07E-06	2.23E-05	-0.762121451	6.35E-08	1.51E-06
Q8NBS9	TXNDC5	-0.223938784	0.035889708	0.104931268	-0.668024891	0.001807725	0.009229264	-1.239719033	4.19E-08	1.10E-06
Q9BPX5	ARPC5L	1.98056912	1.46E-09	5.15E-08	0.971772869	0.001217319	0.006624621	2.771632769	2.14E-12	2.78E-10
O14920	IKBKB	-0.23841737	0.068381146	0.17663877	-0.954726416	0.002279428	0.011115682	-0.856969563	0.003353014	0.012462275
Q9Y3B8;Q9Y3B8-2;Q9Y3B8-3	REXO2	0	1	1	0.905130087	0.000416827	0.002823146	0.718664927	0.007481687	0.024418874
P01034	CST3	0.427946364	0.000533099	0.003344675	-0.684193337	0.00159022	0.008272684	-0.614942835	0.004435112	0.015719538
P42331-4;P42331-6	ARHGAP25	0.436969684	0.00150273	0.007815082	0.729441472	6.13E-07	1.40E-05	0.781115665	1.12E-05	0.000108645
Q96E39	RBMXL1	0.291280147	0.091698799	0.221762664	0.604111817	0.000889666	0.005128783	0.740317056	0.000378197	0.002051264
Q13451	FKBP5	6.12E-17	0.999999989	1	-0.576799462	7.84E-05	0.000716331	-0.7797058	8.01E-06	8.23E-05
Q8WXA9-2	SREK1	0.299082375	0.093317366	0.224598657	0.781923935	9.67E-05	0.000852841	0.881190984	6.87E-05	0.000505329
Q00577	PURA	0.506670381	5.53E-05	0.000510219	0.925204438	5.61E-10	3.14E-08	1.202834399	3.19E-16	1.38E-13
P57088	TMEM33	2.05E-16	0.999999993	1	-1.250247602	0.001144138	0.006290514	-1.286482425	0.00436768	0.015565475
P08758	ANXA5	0.920325907	2.00E-07	3.75E-06	-1.638503651	2.68E-05	0.000306657	-0.955773633	0.014045835	0.041093559
P05109	S100A8	0.257460787	0.225277016	0.449817109	-3.326740007	1.05E-15	4.92E-13	-3.146995454	9.67E-11	6.98E-09
P01857;P01857-1;P0DOX5	IGHG1	0.400672652	0.073516457	0.186086884	0.729315996	0.000251505	0.001870608	1.081399542	8.92E-06	9.06E-05

O43516;O43516-3	WIPF1	2.071992853	7.51E-05	0.000655434	0.767689783	0.007675108	0.029820992	2.336705062	0.000267521	0.001527516
Q14005;Q14005-2;Q14005-3	IL16	0.626143939	1.24E-09	4.56E-08	1.07995548	7.95E-18	5.57E-15	1.13310255	4.27E-11	3.83E-09
Q8NBJ5	COLGALT1	0.379607075	0.000121846	0.000972011	-1.24398794	6.12E-05	0.00059587	-0.772178946	0.007922489	0.025664123
O95865	DDAH2	0.762362116	0.005732382	0.023807279	1.153014621	4.33E-11	3.57E-09	1.791750362	8.48E-08	1.91E-06
P28676	GCA	0	1	1	-2.452467852	2.05E-07	5.42E-06	-2.638888477	3.41E-08	9.13E-07
Q86YV0	RASAL3	0.76036021	1.37E-06	2.01E-05	1.212650618	2.53E-10	1.76E-08	1.717586137	2.64E-11	2.64E-09
Q96S97	MYADM	-9.70E-07	0.998655703	1	-0.99020461	0.006244026	0.025083452	-1.312257793	0.002916482	0.011142678
P47897	QARS1	4.47E-16	0.999999975	1	-0.514867758	0.001634815	0.008457602	-0.508689077	0.00199446	0.008172882
P42785	PRCP	0.395802008	7.48E-05	0.000654345	0.604945506	4.17E-05	0.000430239	0.875315837	4.52E-09	1.59E-07
P40121	CAPG	-0.016178432	0.763691947	1	-2.259951759	1.21E-06	2.49E-05	-3.067040626	3.23E-09	1.22E-07
P22087	FBL	0.563829302	4.09E-07	6.86E-06	0.607029839	8.65E-10	4.58E-08	0.93613589	2.31E-11	2.40E-09
P49189	ALDH9A1	0.561055556	4.85E-06	6.06E-05	0.47337017	0.000500974	0.003282084	0.805765232	4.84E-07	7.96E-06
P46063	RECQL	0.560444568	4.51E-05	0.000429357	0.584374773	3.05E-05	0.000340401	0.896941939	2.23E-07	4.26E-06
Q96B97	SH3KBP1	0.473855571	4.56E-09	1.33E-07	0.648473843	1.74E-07	4.70E-06	0.827073803	1.10E-08	3.43E-07
Q9NZM1;Q9NZM1-6	MYOF	0.573217971	0.005026784	0.021510242	-1.082919114	4.64E-05	0.00046932	-0.794681247	0.000633018	0.00321788
O75695	RP2	7.96E-19	1	1	-0.918425335	0.001696888	0.008714421	-1.108925426	0.001570062	0.006708917
Q9BRR6;Q9BRR6-2	ADPGK	-0.005593006	0.884169214	1	-0.77495899	0.000796434	0.004662218	-1.103153337	1.29E-05	0.00012149
Q96BX8	MOB3A	0.159703967	0.23625764	0.465908524	0.556216024	0.000405446	0.002780598	0.567968268	0.003628199	0.013276144
P51149	RAB7A	7.47E-17	0.999999986	1	-0.947089814	7.23E-09	2.74E-07	-1.079846967	5.29E-11	4.58E-09
P14625	HSP90B1	-0.120505781	0.055728107	0.150919589	-0.533926816	8.27E-07	1.77E-05	-0.910347332	6.64E-14	1.33E-11
P52630;P52630-4	STAT2	4.80E-17	0.999999993	1	-0.886301642	1.57E-06	3.03E-05	-0.669757109	0.000173033	0.001070334
Q96SB3	PPP1R9B	-1.42E-17	0.999999998	1	-0.47341756	0.011325888	0.039581278	-0.875445625	9.39E-06	9.41E-05
O00567	NOP56	0.361327385	0.001576457	0.008138489	0.592747469	6.56E-06	9.89E-05	0.778431564	4.90E-08	1.25E-06
O14974-4	PPP1R12A	0.719298271	2.07E-08	4.97E-07	0.851334826	2.53E-06	4.49E-05	1.260680895	1.63E-10	1.01E-08
Q13951	CBFB	-3.00E-15	0.999999887	1	1.019022071	6.94E-20	9.72E-17	0.591562927	2.60E-06	3.29E-05
O43815	STRN	0.598159296	0.000136474	0.001066977	0.530203805	7.07E-05	0.000661127	0.897544445	1.42E-06	1.99E-05
Q13442	PDAP1	0.204223061	0.082278219	0.203588071	0.525606977	1.70E-05	0.000211124	0.537934068	0.000198532	0.001205109
P08670	VIM	0.729852145	3.48E-06	4.53E-05	-1.118889686	6.29E-10	3.46E-08	-0.550396994	0.003417234	0.012664729
P36957	DLST	0.652288657	0.000216682	0.001578559	1.038323911	3.93E-07	9.43E-06	1.156827787	5.80E-10	2.84E-08
Q96CN7	ISOC1	0.426718348	0.006546027	0.026683574	0.609207726	0.000522389	0.003367305	0.806441444	1.24E-05	0.000119751
P38606	ATP6V1A	0.361058977	0.002651967	0.012653365	-1.011651079	5.47E-07	1.29E-05	-0.752530703	0.000134227	0.000878395
Q9BT09	CNPY3	-0.226102958	0.071961148	0.183334544	-0.726628135	0.000707974	0.004241793	-1.467741695	3.09E-10	1.71E-08
P53602	MVD	0.454946009	0.028004781	0.086542383	1.169457021	7.03E-06	0.000104876	1.440686558	5.76E-06	6.34E-05
P41218	MNDA	0.276706933	0.004450856	0.019398912	-2.245823665	1.16E-07	3.33E-06	-2.104372753	6.30E-07	1.02E-05
P32119	PRDX2	2.21E-15	0.999999925	1	0.767045471	3.15E-06	5.36E-05	0.612055428	0.000112359	0.000762165
O75874	IDH1	0.110857078	0.215795019	0.435953689	-1.397508356	1.58E-09	7.76E-08	-1.47942404	3.65E-09	1.35E-07
P50749	RASSF2	0	1	1	-0.464678827	0.004310399	0.018343067	-0.809828807	2.66E-05	0.000227341
P61626	LYZ	-0.835164001	0.006267064	0.02578491	-3.624359727	5.47E-12	7.31E-10	-4.777228714	2.74E-14	6.47E-12
P51659	HSD17B4	-4.62E-16	0.999999967	1	-0.468762362	0.014521632	0.048017283	-0.84611619	6.82E-06	7.17E-05
Q13492;Q13492-2;Q13492-5	PICALM	0	1	1	-0.86900336	0.000793625	0.00465549	-1.103518596	2.61E-05	0.000224448
Q13363;Q13363-2	CTBP1	0.834772637	6.63E-06	8.06E-05	0.518620008	0.00085911	0.00497716	1.129449474	6.85E-07	1.10E-05
P45954;P45954-2	ACADSB	0	1	1	0.800719325	1.20E-07	3.40E-06	0.620714977	0.000468709	0.002485117

P35443	THBS4	0	1	1	1.759746687	0.001188145	0.006494264	1.85227806	0.000536552	0.002782358
Q96IU4	ABHD14B	0.349376811	0.031070841	0.093717525	0.493358263	0.000183663	0.001426565	0.725884212	0.000223486	0.00131922
Q5SSJ5	HP1BP3	0.024768069	0.684637877	1	0.827513708	3.00E-06	5.16E-05	0.691228087	0.00039163	0.002117196
P00738	HP	0.478431182	0.000263223	0.001856246	-1.523559765	7.19E-07	1.57E-05	-1.058084386	0.000159177	0.001006182
O95218;O95218-2	ZRANB2	0.491863593	0.023053508	0.073629229	0.89319344	9.31E-06	0.000129276	1.002065593	0.000133503	0.000876195
Q9UFN0	NIPSNAP3A	0.332888911	0.020589194	0.067282203	-0.755756656	0.000446262	0.002958199	-0.684017569	0.00047773	0.002522648
Q96HE7	ERO1A	0.231824196	0.01382416	0.048743989	-0.571931958	0.001244216	0.006717292	-0.607134818	0.001243104	0.005568248
Q7L5N7	LPCAT2	-3.60E-19	1	1	-1.693573598	2.05E-05	0.000242809	-1.936336256	1.68E-05	0.000152333
P05164;P05164-2;P05164-3	MPO	-0.217624862	0.246307303	0.480885686	-3.512848337	3.76E-12	5.55E-10	-4.4765151	3.28E-15	9.48E-13
P21281	ATP6V1B2	0.414619881	0.00253603	0.012149512	-1.050894294	1.22E-05	0.000163667	-0.690407885	0.00344843	0.012743986
P20645	M6PR	9.76E-18	0.999999999	1	-0.707367393	0.010608885	0.037702554	-1.182401358	0.000367706	0.002011156
Q15942	ZYX	1.27455195	4.87E-05	0.000460554	1.288685666	4.43E-06	7.13E-05	1.80068391	1.61E-06	2.22E-05
O75563	SKAP2	1.04E-17	0.999999996	1	-0.725807097	9.64E-05	0.000852629	-1.007012306	1.63E-06	2.23E-05
Q8NFW8	CMAS	0.232003718	0.024219187	0.076726734	0.879822646	4.45E-09	1.81E-07	0.93330902	1.40E-06	1.96E-05
O00479	HMGN4	0.493832762	0.067102833	0.173846134	1.891018157	1.06E-06	2.22E-05	2.121481704	1.89E-08	5.50E-07
P53999	SUB1	0.480304477	0.016450401	0.055826867	0.625773001	6.29E-05	0.000605808	1.361300175	2.02E-08	5.71E-07
Q5T1M5	FKBP15	1.40E-17	0.999999994	1	-0.694609324	3.34E-06	5.64E-05	-0.842268319	6.33E-05	0.000473733
P11177;P11177-2	PDHB	0.644547719	0.000480964	0.003083419	0.605350016	0.000150421	0.001215503	0.900615411	3.09E-07	5.46E-06
P42765	ACAA2	0.782282202	0.000121762	0.000972011	0.857185175	2.98E-08	9.94E-07	1.438997854	8.99E-11	6.87E-09
P30040	ERP29	0.208942749	0.042504267	0.120668313	-1.002914353	3.71E-08	1.20E-06	-0.852637381	9.11E-06	9.17E-05
P49902	NT5C2	-1.33E-18	0.999999999	1	-0.683581115	0.001505489	0.00792744	-1.184941335	1.29E-06	1.89E-05
P04792	HSPB1	4.66E-16	0.999999976	1	0.802103855	0.006020305	0.024359214	0.976171138	0.001757148	0.007386846
O75083	WDR1	0.446103776	0.001315404	0.007069701	0.505929202	0.000272385	0.001983814	0.761927767	4.37E-06	5.02E-05
Q6NYC8	PPP1R18	0.801024973	0.004242358	0.018651565	0.877181112	7.74E-05	0.000709338	1.181119202	0.000601173	0.003086654
Q15208	STK38	0.134099761	0.113558318	0.262217832	0.84136334	1.87E-11	1.87E-09	0.754152536	7.07E-10	3.28E-08
P07858	CTSB	0	1	1	-1.995399677	1.47E-08	5.21E-07	-2.367745335	9.28E-11	6.89E-09
Q9NZK5	ADA2	-0.28200125	0.056919937	0.153205875	-0.932486503	1.54E-05	0.000193986	-1.546158839	3.19E-07	5.56E-06
P50395	GDI2	0.129684757	0.137900167	0.306773495	-0.588102562	0.001119288	0.006205204	-0.603986147	0.001652457	0.007014844
O15427	SLC16A3	-0.709316162	0.008339268	0.032526837	-0.861656714	0.000405586	0.002780598	-2.423761291	2.28E-10	1.38E-08
P00492	HPRT1	-1.09E-15	0.999999932	1	-0.929985025	5.85E-08	1.84E-06	-1.000793672	1.44E-07	2.99E-06
Q96CX2	KCTD12	7.44E-16	0.999999958	1	-2.350328243	1.11E-07	3.22E-06	-2.725962233	6.31E-08	1.51E-06
Q8TDZ2;Q8TDZ2-4	MICAL1	1.84E-15	0.999999914	1	-0.523743886	0.002829891	0.013247103	-1.185552121	9.05E-10	4.13E-08
Q9UBR2	CTSZ	-2.66E-05	0.98795026	1	-1.70971459	2.64E-10	1.76E-08	-2.114373602	1.41E-11	1.53E-09
P22392-2	NME2	4.96E-17	0.999999986	1	-0.634819827	8.63E-07	1.83E-05	-0.59791254	0.000529227	0.00276497
P46109	CRKL	0.856169768	3.84E-12	3.15E-10	0.541432063	6.85E-07	1.52E-05	0.987376747	1.45E-12	2.06E-10
P0DOX7	sp|P0DOX7|IGK_HUMAN	0.320389441	0.170930211	0.363163961	1.058089921	8.13E-06	0.000117488	1.184846808	4.06E-05	0.000324862
Q7L591	DOK3	0	1	1	-0.823067814	0.002300946	0.011181718	-1.035866272	0.000169856	0.00105571
O43776	NARS1	4.70E-17	0.999999986	1	-0.56167382	2.95E-05	0.000331419	-0.682606987	1.28E-05	0.00012108
P04839	CYBB	0.420532354	0.20365098	0.418213951	-3.61896285	4.95E-10	2.88E-08	-3.24640089	1.35E-06	1.93E-05
Q6VY07	PACS1	0.128564889	0.141965771	0.313248628	0.721236231	5.17E-12	7.25E-10	0.694197632	0.006768376	0.022543898
P17693;P17693-5	HLA-G	2.70E-16	0.999999977	1	1.011440251	0.008006768	0.030752983	1.193134919	0.001116508	0.00516908
P32456	GBP2	1.211896833	6.61E-11	3.53E-09	-0.618612314	0.001163042	0.00636947	0.692290318	0.008581718	0.027593198

P20839;P20839-3;P20839-5;P20839-6;P20839-7	IMPDH1	0.690099219	1.66E-12	1.50E-10	-0.928012643	5.12E-05	0.000513146	-0.691221295	0.00110252	0.005124057
P25774	CTSS	0.255921583	0.032696631	0.097867845	-1.071958953	9.23E-05	0.000824654	-1.052763938	9.74E-05	0.000687935
Q6P4A8	PLBD1	-7.33E-16	0.99999996	1	-1.867818358	2.31E-08	7.98E-07	-2.038914584	4.87E-06	5.55E-05
P35244	RPA3	0.422414534	0.09112177	0.220973427	0.883970836	0.001488848	0.007862015	1.244645312	1.15E-05	0.000111331
P43405	SYK	-0.215381582	0.057012953	0.153224124	-0.895050426	6.59E-07	1.48E-05	-1.414902841	3.91E-10	2.07E-08
Q9UGI8	TES	0.31163229	0.110157078	0.255872107	0.899587528	1.42E-06	2.81E-05	1.047613789	5.37E-06	6.06E-05
Q9UKD2	MRTO4	-2.04E-16	0.999999988	1	1.141007567	0.000657899	0.004045503	1.155804534	0.000119278	0.00080281
Q06187	BTK	3.20E-16	0.999999959	1	-0.824809805	0.005497992	0.022604649	-1.144898098	0.002144523	0.0086255
Q14012	CAMK1	1.497122263	1.31E-16	2.88E-14	-1.256150783	1.64E-07	4.51E-06	0.961063164	4.88E-05	0.000378767
Q9H4M9	EHD1	0.190160805	0.009898377	0.037528686	0.707576482	9.13E-10	4.74E-08	0.711929778	5.08E-10	2.59E-08
Q5TEC6	NA	0.208321393	0.293872656	0.556196986	0.865126731	0.001957413	0.009848491	1.038473675	0.000133554	0.000876195
Q15555-5	MAPRE2	0.35171325	0.063165247	0.165714777	0.623975428	0.000915247	0.005248167	0.702922006	0.003536038	0.013012219
P29218;P29218-3	IMPA1	0.199214903	0.055981438	0.151373121	0.844469803	1.53E-09	7.66E-08	0.841776693	5.90E-06	6.47E-05
O75436	VPS26A	0.348266872	0.000854173	0.00501134	-0.848731412	1.17E-05	0.000161395	-0.638067111	0.004250219	0.015293725
P83111	LACTB	0.46582303	0.006742523	0.02738956	-0.822766452	0.003241021	0.01482516	-0.91143001	0.000279862	0.001587517
P30536	TSPO	1.08E-15	0.999999978	1	-1.38063226	0.000650588	0.004018166	-1.48588751	0.001721258	0.00725946
Q9BQS8;Q9BQS8-4	FYCO1	5.71E-18	0.999999997	1	0.58486668	0.004273852	0.01826811	1.137425554	7.04E-07	1.12E-05
Q9H0A8	COMMD4	0.309290375	0.037036876	0.107394758	-0.610561854	0.000117836	0.000986306	-0.509155553	0.007096744	0.023427372
P09972	ALDOC	0.274140734	0.039002321	0.112354724	0.680678695	3.17E-05	0.000349686	0.902455773	5.45E-06	6.11E-05
Q9NTX5;Q9NTX5-2;Q9NTX5-6	ECHDC1	0.065647303	0.382584069	0.685811605	-0.512622429	6.21E-06	9.41E-05	-0.622230892	5.97E-05	0.000449318
P01859;P01859-1	IGHG2	0.514798731	0.013953967	0.049054524	1.159860837	1.07E-07	3.12E-06	1.456448671	1.63E-08	4.97E-07
O43852;O43852-3	CALU	0.917389444	0.001153135	0.006343144	0.70487688	0.000775285	0.004567012	1.39511643	2.85E-06	3.51E-05
Q13435	SF3B2	0.826023028	5.47E-07	8.93E-06	0.468885497	0.000938089	0.005351645	0.953279803	9.99E-07	1.51E-05
Q53H82	LACTB2	0	1	1	-1.030932351	0.001467482	0.007773867	-0.924567649	0.001636387	0.006957992
Q9UK45	LSM7	0.043092149	0.599948365	0.99315396	0.583976546	0.001121982	0.006205204	0.535379808	0.003105114	0.0117084
P58876	H2BC5	0.502661661	0.002956099	0.013805568	0.583014872	0.006889797	0.027171575	0.704300365	1.04E-05	0.000102244
O43670;O43670-2;O43670-4	ZNF207	0.572722202	0.05019126	0.138489621	1.422517337	8.04E-09	3.01E-07	1.207878994	0.000314035	0.001773617
Q5VVQ6;Q5VVQ6-2	YOD1	0	1	1	0.817794533	0.001054677	0.005914627	0.638796674	0.010672714	0.032930774
O95834;O95834-2;O95834-3	EML2	0.025499477	0.649775747	1	0.679039937	1.38E-05	0.0001793	0.600164371	0.000148289	0.000958344
P08236;P08236-2	GUSB	0.225958499	0.022299363	0.071740468	0.564616289	5.54E-05	0.000551254	0.665752969	1.00E-06	1.51E-05
O00182;O00182-2;O00182-3;O00182-6	LGALS9	1.165906773	6.91E-10	2.80E-08	-1.372330798	4.77E-08	1.52E-06	-0.618158707	0.00902992	0.028714481
Q30154	HLA-DRB5	0.641862194	0.056381282	0.152220826	-2.34358523	6.12E-07	1.40E-05	-2.079006578	4.59E-06	5.26E-05
Q93091	RNASE6	-0.61345481	0.000154987	0.00118801	-1.912495493	2.17E-07	5.58E-06	-2.371764825	1.31E-07	2.79E-06
Q8TCT9;Q8TCT9-5	HM13	0	1	1	-1.071086434	0.002151857	0.010585624	-1.069571814	0.002250449	0.008994872
Q96QR8	PURB	0.373472917	0.020673685	0.067495752	0.510620887	0.001469383	0.007773867	0.585041892	0.000634163	0.00321788
Q9BUH6	PAXX	0.901010168	0.000480942	0.003083419	1.558370617	1.21E-13	3.09E-11	2.419797765	2.19E-09	8.88E-08
P17480;P17480-2	UBTF	-3.50E-18	0.999999998	1	1.098088735	8.88E-08	2.65E-06	0.806681816	0.000120886	0.000809437

Q8TBX8;Q8TBX8-3	PIP4K2C	6.84E-18	0.999999999	1	1.28737655	4.13E-10	2.51E-08	1.047269307	3.85E-08	1.02E-06
P20933	AGA	-4.57E-17	0.999999992	1	1.253960943	1.55E-11	1.74E-09	0.940211276	2.82E-07	5.12E-06
Q05315	CLC	0.684392794	0.07751609	0.194396681	2.285828334	2.91E-06	5.07E-05	3.009126969	2.74E-07	5.04E-06
Q2M2I8;Q2M2I8-2	AAK1	0	1	1	0.711016879	0.000284627	0.002062258	0.724550056	0.001405379	0.006136796
Q15637;Q15637-2;Q15637-3;Q15637-4;Q15637-6	SF1	0.230067305	0.22335028	0.44670056	0.498347462	0.000129408	0.001073554	1.026345732	9.57E-05	0.000679444
P48960	ADGRE5	1.17E-17	0.999999996	1	-0.893052348	0.000384716	0.002663568	-0.607694466	0.016921702	0.04831053
A0A075B6P5;A0A087WW87;P01614;P01615	IGKV2-28;IGKV2-40;IGKV2D-40;IGKV2D-28	0	1	1	1.080332829	4.30E-06	7.01E-05	1.045782022	0.000492628	0.00258555
Q9NY12;Q9NY12-2	GAR1	0.821543197	5.56E-05	0.000512212	0.612591766	0.001219082	0.006624621	1.161462363	4.35E-05	0.000344475
Q92572	AP3S1	0	1	1	-0.502166384	0.003641414	0.016104928	-0.774190673	0.000163525	0.001026179
P49419;P49419-2	ALDH7A1	-3.56E-16	0.999999967	1	-0.611921342	0.009260854	0.034134149	-1.078547967	0.000118224	0.000797779
P22830;P22830-2	FECH	0.207095679	0.177192749	0.374569325	1.009191233	6.08E-07	1.40E-05	1.005365195	3.37E-05	0.00027915
O00602	FCN1	-0.51988107	0.067198619	0.173966469	-3.877856884	7.47E-08	2.25E-06	-3.765314423	1.21E-06	1.80E-05
Q9BV40	VAMP8	-7.44E-19	0.999999999	1	-0.942467802	0.004194505	0.01809445	-1.207600591	0.000245573	0.001424106
Q14847	LASP1	1.979487162	5.96E-08	1.33E-06	1.590209025	3.22E-11	2.92E-09	2.906340808	6.51E-10	3.13E-08
P56192	MARS1	0	1	1	-0.549327166	2.01E-06	3.76E-05	-0.755986837	7.16E-07	1.13E-05
Q6RW13	AGTRAP	-0.117564639	0.482509206	0.828299641	-0.692618941	0.005272169	0.021864371	-2.105909587	2.37E-06	3.07E-05
Q9UBF2	COPG2	3.14E-16	0.999999969	1	-0.850041762	7.11E-05	0.000662361	-1.051059498	0.000100556	0.000706067
O00151	PDLIM1	0.424824744	0.225881006	0.450484405	1.178097443	0.002237836	0.01093187	2.004588267	1.58E-05	0.000145649
P09668	CTSH	0.582742229	0.000881574	0.005120973	-1.689500974	1.55E-06	3.01E-05	-1.452460139	7.73E-06	7.97E-05
Q6UW68	TMEM205	5.98E-17	0.999999997	1	-1.051693301	0.003525664	0.015692002	-1.3487393	0.001556953	0.006685889
Q8IYJ3	SYTL1	0.405017975	0.000191528	0.001412823	0.989097548	1.93E-09	9.35E-08	0.966763539	1.42E-07	2.97E-06
O95400	CD2BP2	0.302193069	0.035384568	0.103798657	0.671998888	2.36E-06	4.32E-05	0.630580975	0.001024876	0.004806188
Q9BT23	LIMD2	9.20E-19	0.999999999	1	0.932458634	1.53E-09	7.66E-08	0.721524315	0.000436296	0.002332296
P35813;P35813-3	PPM1A	0.136889722	0.207335883	0.423561022	0.648542003	1.20E-05	0.000163147	0.694579392	0.000396135	0.002130762
Q9HC16	APOBEC3G	0.329732118	0.100494083	0.238293299	1.813604904	3.41E-11	2.98E-09	2.130712495	5.09E-09	1.74E-07
O94925	GLS	0.628566406	0.003974522	0.017694652	1.132337133	6.58E-09	2.53E-07	1.493643294	3.09E-07	5.46E-06
Q04941	PLP2	-0.803634774	0.019159581	0.063552854	-1.165661162	0.000243631	0.001826584	-2.330212141	7.73E-08	1.79E-06
P27816	MAP4	0.278902501	0.136620067	0.304502121	0.643735076	0.006878059	0.027163489	1.274725471	2.98E-06	3.65E-05
Q9BVG4	PBDC1	0.220766618	0.087673998	0.214233205	0.531611384	2.70E-06	4.77E-05	0.72053968	1.26E-05	0.00012077
Q8TCE6	DENND10	0.299036189	0.00746699	0.029783491	-0.750152393	7.96E-06	0.000115675	-0.753019674	3.87E-06	4.54E-05
P17174	GOT1	0.119997326	0.341470174	0.623523477	0.636190346	0.00140747	0.007503265	0.542702397	0.012316125	0.03711983
Q9HAU5	UPF2	0.302426416	0.05724124	0.153718669	0.543483995	0.001959864	0.009848491	0.545479627	0.006065821	0.020519534
O15347	HMGB3	-3.44E-17	0.999999989	1	-1.123475474	2.55E-05	0.000294424	-1.001349054	0.000206528	0.001247814
Q9NTM9	CUTC	2.75E-15	0.999999923	1	1.046191082	4.20E-10	2.51E-08	0.869657693	3.41E-05	0.000281051
Q5VSL9;Q5VSL9-2	STRIP1	0.41180855	0.00179596	0.009085442	0.51239881	0.000239813	0.001812201	0.614920288	4.53E-05	0.000355936
Q9Y2I7	PIKFYVE	1.805488733	6.29E-08	1.39E-06	-0.480008743	0.009901826	0.035825445	0.847127562	0.001146808	0.005245437
P17612	PRKACA	0.702155369	5.82E-05	0.000531755	-1.184921703	7.03E-08	2.17E-06	-0.819989972	0.002038991	0.008289981
P98175;P98175-2;P98175-5	RBM10	0.282177702	0.040690214	0.116645281	0.601314486	0.000828366	0.004824332	0.997823881	0.00180824	0.007540356
Q5VTL8	PRPF38B	0.363410624	0.001901871	0.009541143	0.497042583	2.33E-05	0.000272989	0.611261235	9.69E-07	1.47E-05

Q8TBC4;Q8TBC4-2	UBA3	0.137654155	0.172797084	0.366595979	0.774841247	3.23E-08	1.06E-06	0.758087111	5.48E-06	6.11E-05
Q10567-3	AP1B1	0.013421588	0.780298011	1	-0.689660808	4.93E-05	0.000495909	-0.730764472	0.000357703	0.001964717
O94905	ERLIN2	-3.80E-19	1	1	-1.139966305	0.000645073	0.004001739	-2.233045683	1.64E-06	2.23E-05
Q8WVT3	TRAPPC12	6.89E-16	0.999999967	1	-0.65221463	0.002138791	0.010572328	-0.949678219	1.38E-05	0.000129235
Q9HBH0	RHOF	0	1	1	1.17605695	0.000134678	0.0011042	1.188275995	3.84E-06	4.53E-05
Q5R3I4	TTC38	0.370161979	0.076001012	0.191140918	1.254675098	3.50E-06	5.87E-05	1.106755593	0.00091763	0.004342448
Q05209	PTPN12	0.341905629	0.013243039	0.047214311	0.846701558	1.21E-05	0.000163468	0.643210994	0.001854327	0.007683479
Q15904	ATP6AP1	3.64E-16	0.99999999	1	-1.104694833	0.000392269	0.002709169	-1.170783095	0.003667812	0.013402214
O95721	SNAP29	0.842985957	0.004920671	0.021158886	0.635407195	0.006093194	0.024547868	0.958721882	0.00115022	0.005251794
P53634	CTSC	0.368904846	0.005770797	0.023938624	1.773479307	4.39E-16	2.46E-13	2.136217223	1.14E-18	1.48E-15
O14618	CCS	0.444072049	0.034762649	0.102571632	0.646926163	4.11E-05	0.000426245	0.563965146	0.007325205	0.023998592
P30533	LRPAP1	-0.246859629	0.058625578	0.156517776	-0.639162073	0.00082929	0.004824332	-1.310707035	2.27E-09	8.93E-08
Q8IV04	TBC1D10C	0.37087441	0.065329506	0.170252651	1.389867419	6.45E-09	2.51E-07	1.509432395	4.57E-08	1.18E-06
Q9UEW8;Q9UEW8-2	STK39	-0.381377484	0.076630723	0.192313117	1.116504426	5.04E-06	7.93E-05	0.757845977	0.003698598	0.013495728
Q99733;Q99733-2	NAP1L4	0.95167726	0.00025526	0.001807326	0.667775142	0.000107377	0.000925392	1.520421049	0.000124868	0.000833952
Q9NYB0	TERF2IP	0.153075466	0.229682425	0.455489444	0.628502314	1.74E-12	3.25E-10	0.598775933	0.000119881	0.000804784
P24158	PRTN3	-0.93138002	0.007755905	0.030692842	-3.457445174	1.73E-11	1.85E-09	-5.075542177	6.14E-13	1.06E-10
O43399;O43399-5;O43399-7	TPD52L2	1.345674079	2.05E-06	2.88E-05	-0.823435075	3.72E-06	6.17E-05	0.52808817	0.010819733	0.033305292
Q9C0A0;Q9C0A0-2	CNTNAP4	0.687438316	0.111694251	0.258628247	2.297022812	1.77E-06	3.40E-05	3.328673138	3.53E-11	3.31E-09
Q8WUD4	CCDC12	0.796273207	0.005003543	0.021462885	0.774075265	0.000340297	0.002397467	1.436558666	1.48E-05	0.000138753
A0A0A0MRZ8;P04433	IGKV3D-11;IGKV3-11	0.710797197	0.001990393	0.009926629	1.294328993	7.14E-08	2.17E-06	1.747674029	4.97E-08	1.25E-06
P01619	IGKV3-20	0.57201441	0.027984788	0.086542383	0.966044984	0.000236842	0.001794875	1.364708131	2.21E-05	0.000194188
A0A075B6H7;A0A0C4DH55;P01624	IGKV3-7;IGKV3D-7;IGKV3-15	0.87304851	0.000742962	0.004432629	1.321816841	3.15E-06	5.36E-05	2.108791169	6.43E-12	7.95E-10
P50750;P50750-2	CDK9	0.338092039	0.029798138	0.090654214	0.644732354	0.011707534	0.040528302	0.765684921	0.000487117	0.002561801
P30043	BLVRB	0.258219076	0.024430709	0.077321551	-1.759497667	6.59E-07	1.48E-05	-1.717614149	1.91E-06	2.54E-05
Q14118	DAG1	0.139262304	0.511097691	0.867242761	1.545448991	1.52E-05	0.000192793	1.843515994	3.67E-05	0.000298661
Q9BPW8	NIPSNAP1	0	1	1	0.822211587	3.43E-05	0.000374033	0.78603927	0.000838267	0.004010716
P16150	SPN	3.55E-16	0.999999972	1	1.382121293	6.31E-05	0.000605808	1.728552134	1.39E-06	1.96E-05
P04908;Q7L7L0	H2AC4;H2AW	1.100104835	0.006296211	0.025874638	1.537944339	0.000114409	0.00096627	2.531137303	1.91E-08	5.50E-07
P15531;P15531-2	NME1	-0.238508125	0.21668141	0.436675949	-0.621584244	0.004251855	0.018208161	-1.393147032	4.32E-06	5.01E-05
P05090	APOD	2.64E-16	0.999999982	1	1.057906977	0.007678587	0.029820992	1.263860399	0.009703188	0.030408785
Q9NX24	NHP2	0.429049315	0.012092934	0.043913167	0.481149931	0.000173552	0.001374688	0.852601802	6.64E-06	7.04E-05
Q15427	SF3B4	0.766743389	0.029351425	0.089682083	0.850429419	0.000177909	0.001401281	1.427908472	0.000341558	0.001896085
O94966;O94966-3;O94966-4;O94966-5;O94966-6;O94966-7	USP19	0	1	1	1.628075147	0.003106377	0.01430763	1.452302039	0.00116573	0.005313275
Q9UDT6;Q9UDT6-2	CLIP2	0	1	1	1.956883039	6.18E-05	0.000599317	1.992888677	0.000228717	0.001341323
P08246	ELANE	-1.52499677	3.18E-05	0.000311942	-3.722049913	3.15E-10	2.01E-08	-5.706215737	3.42E-14	7.40E-12
Q8ND56;Q8ND56-2;Q8ND56-3	LSM14A	4.70E-16	0.999999969	1	0.831788791	2.88E-06	5.05E-05	0.560240693	0.007293741	0.023925681

Q96M27;Q96M27-2;Q96M27-3	PRRC1	0.336633483	0.001039069	0.005834011	0.533008085	0.002141613	0.010572328	0.520610077	0.009250168	0.029271542
Q13315	ATM	0.276289486	0.143462942	0.315761756	0.6969864	0.000249894	0.001863571	0.638879749	0.013457176	0.039594272
Q14644	RASA3	0	1	1	1.241604218	1.23E-07	3.46E-06	0.88688024	0.000844541	0.004033304
P51668	UBE2D1	0	1	1	-1.04293535	7.66E-05	0.000704136	-1.456663329	6.02E-08	1.46E-06
Q96F07-2	CYFIP2	0.714129186	3.77E-05	0.000364393	0.995949947	2.54E-09	1.15E-07	1.425290807	1.43E-10	9.24E-09
Q16799	RTN1	-0.607224724	0.013014148	0.046539438	-1.209784434	0.000162343	0.0013006	-2.198895976	2.69E-08	7.29E-07
Q16270;Q16270-2	IGFBP7	0.027031711	0.698367887	1	0.940854023	0.000107588	0.000925392	0.913943797	2.72E-05	0.000230184
P22033	MMUT	0.538190205	0.022498566	0.072315355	0.843406112	0.000242427	0.00182243	1.212061756	8.52E-06	8.68E-05
Q96I59	NARS2	0.518785078	0.084940781	0.209148879	-0.98272328	0.002015072	0.010071766	-0.729700966	0.001547525	0.006656409
O95197-3	RTN3	-0.356399174	0.021663957	0.070144272	-1.209394937	5.36E-06	8.40E-05	-2.141291994	1.03E-08	3.28E-07
P01834	IGKC	0.597652311	0.046988008	0.131491837	0.634754635	0.009671029	0.035217617	1.090831873	0.003103562	0.0117084
Q8IWA5;Q8IWA5-3	SLC44A2	0.623901434	0.009895657	0.037528686	0.724642686	0.009223622	0.034079683	1.145412818	0.000150011	0.000967066
O14966	RAB29	0.474590426	0.024763247	0.078239435	1.14338881	0.001905051	0.009624799	1.64838349	3.19E-06	3.87E-05
Q15149-3	PLEC	0.0753402	0.442139822	0.769869141	-1.00585214	8.55E-06	0.000121131	-1.060997941	1.27E-05	0.000120813
Q15149-4	PLEC	3.21E-20	1	1	-0.789710402	0.000186465	0.001444332	-1.05987127	0.000162195	0.001020297
P20290;P20290-2	BTF3	0.743713827	0.005956711	0.024622933	0.519528191	0.008938059	0.033461037	1.081539008	0.000664543	0.003332981
Q9UKD1	GMEB2	0.602438707	0.003297444	0.015119361	0.590667765	0.008520723	0.03224306	0.838938074	0.001678281	0.007112845
P05114	HMGN1	0	1	1	1.147966455	0.001069331	0.005961042	1.06498665	0.003305583	0.012356698
Q9UFW8	CGGBP1	0.91940737	8.65E-05	0.000737014	0.837092052	0.000304573	0.002178626	0.68225497	0.010366174	0.032164795
B9A064;P0DOX8	IGLL5	0	1	1	1.131546539	0.008596605	0.03248636	0.986223773	0.01261256	0.037681337
Q14011	CIRBP	0	1	1	1.008100535	0.00050222	0.003282573	1.037159702	0.000153135	0.000979911
